# The effectiveness of nature‐based interventions on mental health: A systematic review

**DOI:** 10.1111/aphw.70164

**Published:** 2026-07-02

**Authors:** Darlene Heinen‐Stach, Erika Herbold, Carla L. Hendricks, Niklas Zuch, Michael A. Rapp, Michèle D. Birtel, Mira Tschorn

**Affiliations:** ^1^ Social and Preventive Medicine, Department of Sports and Health Sciences, Intra Faculty Unit “Cognitive Sciences”, Faculty of Human Science and Faculty of Health Sciences Brandenburg Research Area Services Research and e‐Health Potsdam Brandenburg Germany; ^2^ German Center for Mental Health (DZPG), partner site Berlin/Potsdam Potsdam Germany; ^3^ Institute for Lifecourse Development University of Greenwich London UK; ^4^ German Center for Mental Health (DZPG), partner site Berlin/Potsdam Berlin Germany; ^5^ Division of Clinical Psychological Intervention and Division of Health Psychology Freie Universität Berlin Berlin Germany

**Keywords:** mental health, nature, nature‐based interventions, systematic review, well‐being

## Abstract

As mental health challenges continue to rise globally alongside increasing urbanization and treatment barriers, nature‐based interventions (NBIs) may provide an accessible, affordable, and low‐risk alternative to conventional mental health treatments. This systematic review synthesizes quantitative evidence on the impact of NBIs on mental health outcomes, following PRISMA 2020 guidelines. Randomized controlled trials with a nature‐based intervention group and a non‐nature control group, reporting at least one mental health outcome, were included. Of 10,113 identified records, 47 trials met the inclusion criteria. Most interventions involved horticultural therapy, green exercise, or nature walks. Seventeen studies reported significantly greater improvements in mental health, particularly depression, anxiety, and stress in NBI groups compared to controls. NBIs showed no evidence of inferiority to established treatments such as cognitive behavioral therapy or art therapy. However, studies varied widely in design, intervention type, duration, and outcome measures, and many showed moderate to high risk of bias. While initial evidence supports the effectiveness of NBIs, future research should prioritize long‐term studies, higher methodological quality, and detailed subgroup analyses to better understand the specific conditions under which NBIs are most beneficial, as well as test the added benefits of combining NBIs with established treatments.

## INTRODUCTION

Mental health disorders are increasingly recognized as a significant public health challenge worldwide. Poor mental health is one of the leading contributors to the global burden of disease (James et al., 2018). Mental health problems are rising worldwide and are projected to continue increasing in the future (Wu et al., 2023). Beyond genetic and psychological influences, social and environmental conditions, such as exposure to natural or urban environments, play a crucial role in mental well‐being. Evidence shows that spending time in nature is positively associated with improved mental health outcomes, including reduced symptoms of depression and anxiety, as well as physical health outcomes, such as better sleep (White et al., 2023). In contrast, increased modern urban living and climate change, characterized by reduced access to green spaces and increased environmental stressors, are linked to poorer physical and mental health (Maas et al., 2009; Paykel et al., 2000; Srivastava, 2009).

Nature‐based interventions (NBIs) may be effective in counteracting negative trends in mental health (Shrestha et al., 2023), with additional benefits for urbanization, in line with the Sustainable Development Goals (SDGs) established by the United Nations. The SDGs define global priorities for people and the planet. In particular, the SDG 3 aims to ensure healthy lives and promote well‐being for all. Interventions that involve bringing people into green spaces, such as NBIs, may not only enhance mental health but also increase access to nature for urban residents who do not have green space nearby and would not actively seek it out themselves. This systematic review synthesizes quantitative studies that examine the effects of NBIs on mental health outcomes.

## NATURE AND MENTAL HEALTH

Previous research suggests that contact with nature has positive effects on mental health (Liu et al., 2023), while urbanization and, with this, a lack of contact with nature have negative effects on mental health (Bratman et al., 2019; Frumkin et al., 2017; Maas et al., 2009). Contact with nature can be defined by geographic indicators, such as the extent of green or blue spaces within specific areas. Green spaces include several types of vegetation and can be interpreted in two ways. The first one refers to predominantly natural or rural landscapes, such as farmland, mountains, or forests. The second interpretation refers to managed or designed urban vegetation, such as gardens or parks, which are embedded in built environments and often shaped by human planning and maintenance. Blue spaces can be defined as all natural surface waters, such as rivers or oceans. Both blue and green spaces, may provide positive effects on mental health (Shrestha et al., 2023). However, there have been calls to broaden this definition, as contact with nature can be experienced on different dimensions such as intrapersonal, interpersonal, interactional, or temporal. For example, intrapersonal contact refers to an individual's subjective experience of nature (e.g., feeling restored while sitting in a park), interpersonal contact involves shared experiences in nature (e.g., social interactions during group walks), interactional contact describes direct engagement with natural elements (e.g., gardening or touching water), and temporal dimensions refer to the duration, frequency, or timing of nature exposure (e.g., daily short visits vs. occasional longer stays) (Shrestha et al., 2023; White et al., 2023).

Modern living environments, such as urban areas, offer essential health‐promoting benefits, such as employment opportunities, access to education (Patel et al., 2018) as well as cultural opportunities and governmental services (Hogan et al., 2016). On the other hand, certain aspects of urban living are associated with negative mental health, for example, limited engagement with and limited access to nature (Maas et al., 2009; Paykel et al., 2000; Srivastava, 2009). Furthermore, urban environments are characterized by high levels of sensory stimulation, such as noise, dense crowds, and constant movement, that intensely capture attention. In addition, navigating such environments often requires individuals to exert effortful, goal‐directed attention (e.g., focusing on traffic or avoiding collisions), which can lead to mental fatigue. In contrast, natural environments typically contain stimuli that are gently engaging rather than overwhelming. These stimuli tend to draw attention in an effortless, involuntary way, thereby reducing the demand on controlled attentional processes.

According to attention restoration theory (ART) (Kaplan & Kaplan, 1989), natural settings promote psychological restoration because they provide what the authors describe as “soft fascination,” that is, stimuli that subtly attract attention without requiring mental effort. This allows for a recovery of the brain's directed‐attention capacity, which is depleted by tasks that demand prolonged focus and cognitive control. In this sense, natural environments offer the specific conditions needed to recover from attentional fatigue and support cognitive functioning. Beyond ART, additional theoretical frameworks help explain the positive influence of nature on mental health. Contact with nature is increasingly understood as a valuable resource for promoting psychological well‐being, not only in terms of acute stress reduction but also regarding long‐term prevention, emotional stabilization, and recovery from psychological strain. Biophilia theory (Wilson, 1984) posits an innate human tendency to seek connections with nature and other forms of life, suggesting that nature exposure meets deep evolutionary needs. Stress recovery theory (Ulrich, 1983) highlights that natural settings elicit immediate physiological and emotional responses that counteract the effects of acute stress. Together, these models emphasize that natural environments can foster calmness, enhance mood, restore cognitive resources, and reduce physiological stress markers such as cortisol levels and blood pressure. A growing body of empirical evidence supports these claims. Studies have consistently shown that exposure to natural settings, whether through green spaces, blue spaces, or immersive nature experiences, is associated with improved mood, enhanced cognitive performance, and measurable reductions in stress indicators (Antonelli et al., 2019; Bowler et al., 2010; Corazon et al., 2019; Frumkin et al., 2017; Twohig‐Bennett & Jones, 2018).

In addition, the positive effects of contact with nature may encourage people to take climate action (in line with the UN SDG13). NBIs have the potential to increase nature connectedness (Keenan et al., 2021), which in turn is suggested to motivate pro‐environmental behavior (PEB) (Mackay & Schmitt, 2019; Martin et al., 2020; Whitburn et al., 2020). Contact with nature is therefore able to synergistically promote both SDG 13 (climate action) and SDG 3 (health and well‐being).

## NBIs

NBIs are defined as programs or activities in which individuals engage with or in a natural environment, with the aim of improving their health and well‐being (Taylor et al., 2022). Although the Oxford Languages definition of “nature” includes “the phenomena of the physical world collectively, including plants, animals, the landscape, and other features and products of the Earth, as opposed to humans or human creations” (n.d.), animal‐assisted interventions are structured, goal‐oriented activities incorporating domestic animals into healthcare and social work settings (Beetz 2017; Fine 2019). NBIs, meanwhile, can be defined as activities conducted within horticultural environments and/or wild natural settings (Annerstedt & Währborg, 2011; Hansen et al., 2017).

NBIs can generally be divided into two categories: first, those that modify people's environment, such as creating parks in urban areas, and second, those that change people's behavior, such as green exercise programs. NBIs can involve green spaces, blue spaces, or a combination (Shrestha et al., 2023). NBIs can be performed in several ways. They can be classified into directly interacting with nature, acting in nature, and interacting with other people in nature (Richardson et al., 2021). Examples of interventions in nature are nature‐based walking, green exercise, or other physical activities in nature, such as hiking or surfing (Ma et al., 2024). Nature provides the space, but the focus is on the activities. On the other hand, green gardening and horticultural therapy are activities in which people interact directly with nature (Gray et al., 2024). Thus, compared to the type of intervention already mentioned, nature itself is at the center of attention. Experiencing these programs with other individuals is the third type of intervention. This type offers new opportunities, especially considering feelings of loneliness and self‐worth (Borgi et al., 2019). Given the diversity of intervention types and formats, it is important to consider the underlying mechanisms through which NBIs are expected to influence mental health.

As urban environments are often linked to negative mental health outcomes, the potential of natural environments to support mental well‐being is attracting increasing interest due to their potential health benefits (White et al., 2023). Standard treatments, such as psychotherapy, can be time intensive, involve long waiting periods, and depend on the compatibility between therapist and patient. Antidepressant medications are often associated with various side effects, including weight change, sexual dysfunction, and gastrointestinal symptoms (see, e.g., Kearns et al., 2022; Riediger et al., 2017 for detailed discussions). In contrast, “natural” therapies, such as meditation, physical activity, and spending time in nature, are more accessible, more cost‐effective, and less complex than conventional treatments like medication or psychotherapy (Nejade et al., 2022).

NBIs therefore may be an effective and efficient way of promoting good health and well‐being (in line with the UN SDG 3). Another reason for the growing interest may be the increasing attention to environmental sustainability. In the context of climate change, protecting natural resources has become an urgent global priority, further elevating the relevance of NBIs within both health and environmental discourses. NBIs may contribute to environmental sustainability by promoting the use, conservation, and appreciation of natural environments, which can, in turn, support pro‐environmental attitudes and behaviors as well as the preservation of green and blue spaces (Shrestha et al., 2023).

Considering nature as a therapeutic intervention, some studies have shown improvements in the treatment and prevention of common mental health problems. NBIs may lead to reductions in depression and anxiety scores and increase well‐being and positive affect (Keenan et al., 2021; Shanahan et al., 2019). Interventions with direct experiences in nature are generally more effective than virtual exposures (Browning, Shipley, et al., 2020). However, virtual nature interventions, such as immersive virtual reality forest experiences, especially in contexts where access to real nature is restricted, may also improve mental health (Browning, Mimnaugh, et al., 2020). A recent systematic review by Spano et al. (2023) synthesized 59 studies examining the psychological and psychophysiological effects of virtual nature experiences, such as immersive virtual environments or 360‐degree videos of natural settings. The findings suggest generally positive effects on mood, stress, and perceived restorativeness, although outcomes such as cognitive performance, nature connectedness, and behavioral intentions remain understudied. Most included studies were of high or very high methodological quality yet often used heterogeneous measures, unclear sampling procedures, and limited statistical power. Future research is needed to determine optimal exposure parameters and to identify which individuals may benefit most. Although not a replacement for real‐life exposure, virtual nature may offer complementary pathways to enhance mental well‐being—particularly in contexts with limited access to natural environments.

### Conceptual framework

**FIGURE 1:**
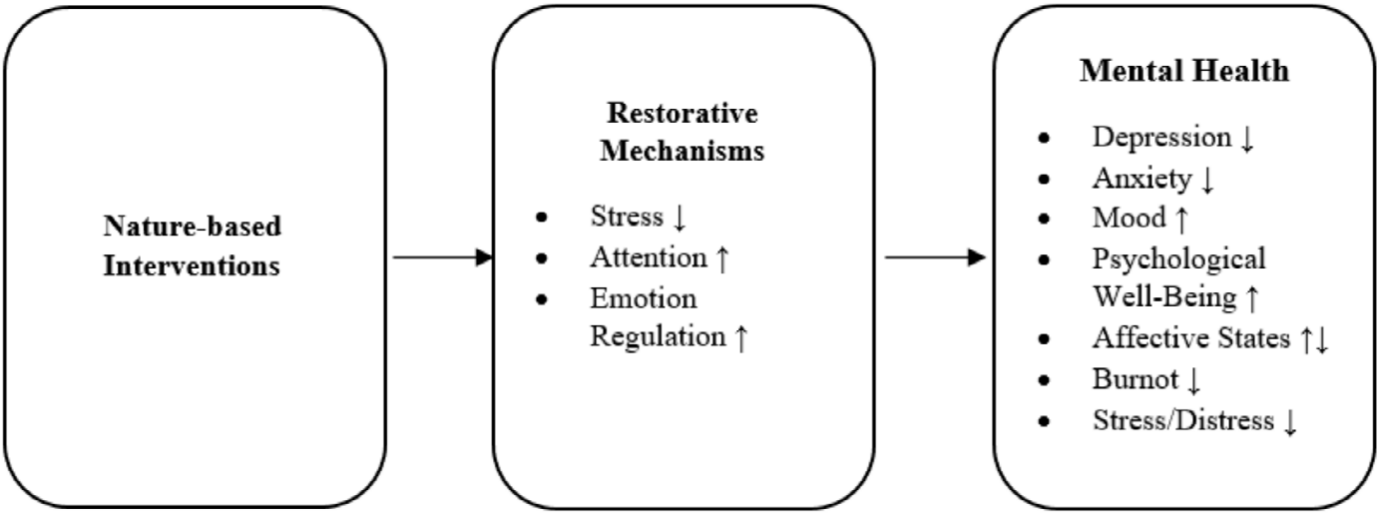
How NBIs influence mental health: A conceptual framework. Adapted from stress reduction theory (Ulrich, 1983), attention restoration theory (Kaplan & Kaplan, 1989), and the biophilia hypothesis (Wilson, 1984).

This review is guided by a conceptual framework that integrates three established theoretical perspectives on the benefit of nature exposures: stress reduction theory, which posits that natural environments elicit positive physiological responses that reduce stress (Ulrich, 1983); ART, suggesting that natural environments facilitate the recovery of cognitive resources (Kaplan & Kaplan, 1989); and the biophilia hypothesis, which argues that humans possess an innate affinity for nature that supports mental health (Wilson, 1984). These theoretical mechanisms are assumed to explain the impact of NBIs on various domains of psychological health. The mechanisms are visualized in Figure 1.

## THIS SYSTEMATIC REVIEW

An umbrella review of 64 systematic reviews (19 with meta‐analyses) published up to 2022 (Shrestha et al., 2023) demonstrated that there is particularly strong evidence for NBIs that include immersive experiences (e.g., forest bathing, horticultural therapy), social interactions, active engagement, and co‐design. Previous systematic reviews have focused on a specific NBI (Kamioka et al., 2014; Kotera et al., 2022), in a specific setting such as rural areas (Batterham et al., 2022) or workplace (Gritzka et al., 2020), exposure to nature rather than interventions (Bowler et al., 2010), or specific populations such as individuals with mental health conditions (Cipriani et al., 2017). Additionally, Rueff and Reese (2023) synthesized randomized trials comparing ecotherapy with cognitive behavioral therapy (CBT) in relation to depression and anxiety, thereby focusing on a narrow comparator and outcome scope. In contrast, our systematic review extends the existing literature in several ways: It (1) considers evidence published until 2025, (2) includes only randomized controlled trials (RCTs) (excluding quasi‐experimental designs) and (3) encompasses a broader range of intervention types, comparator conditions, and mental health outcomes. By doing so, it enables a more comprehensive synthesis and facilitates implementation‐relevant conclusions across diverse clinical and community contexts.

Mental health can be operationalized based on objectively measurable aspects (e.g., physiological indicators of stress) and subjectively experienced states (e.g., emotional well‐being, life satisfaction). In this review, we focused on studies assessing mental health outcomes using validated psychometric scales, capturing a spectrum from mild symptoms to clinically relevant levels of impairment.

To ensure conceptual clarity, we briefly define the key mental health outcomes examined in this review: Depression and anxiety refer to clinically relevant affective disorders characterized by persistent sadness, loss of interest (depression), or excessive worry and physiological arousal (anxiety) and were typically assessed with standardized symptom‐based questionnaires (e.g., PHQ‐9, Yeung et al., 2008; BDI, Beck et al., 1988, 1996; GAD‐7, Spitzer et al., 2006). Stress was conceptualized more broadly and included both perceived stress and psychological distress, often measured via self‐report scales such as the PSS‐10 (Leung et al., 2010). Psychological well‐being was operationalized using various instruments capturing positive mental health, satisfaction with life, and general functioning. In contrast to diagnostic outcomes, well‐being and mood‐related variables (e.g., affect) represent nonclinical, dimensional indicators of psychological functioning. This review therefore includes both clinical and subclinical mental health outcomes, reflecting the diverse conceptual landscape of NBI research.

With this systematic review, our primary objective was to explore the effectiveness of NBIs on mental health outcomes in adults aged 18 and older. We include interventions encompassing programs, activities, or strategies engaging participants with elements of nature (e.g., plants, water, or wildlife) to promote mental health and well‐being outcomes. Specifically, it aimed to (a) to identify intervention strategies (e.g., activities) using nature, defined as incorporating plants, vegetation, or natural spaces, to enhance health outcomes in adults; (b) to determine the types of health effects associated with these interventions in adults; and (c) to examine pathways linking specific forms of natural exposure to health outcomes.

Our secondary objectives were to understand:

1. What types or subgroups of nature‐based interventions (e.g., forest bathing, green exercise) improve mental health outcomes? (RQ1)
2. How do nature‐based interventions, compared to other therapeutic interventions (e.g., CBT, mindfulness‐based therapy) or no intervention, improve mental health? (RQ2)
3. Which specific mental health outcomes (e.g., reduction in depression, anxiety, or stress; improvement in well‐being or mood) are influenced by nature‐based interventions? (RQ3)
4. How robust are the methodologies, and what is the overall quality of studies evaluating the effectiveness of nature‐based interventions for mental health? (RQ4)
5. How do frequency, duration, and intensity of nature‐based interventions influence their effectiveness on mental health? (RQ5)
6. Does the setting (urban green spaces vs. natural wilderness) and group‐based versus individual interventions affect outcomes? (RQ6)
7. Does including physical activity (green exercise) in the intervention method affect outcomes? (RQ7)

We synthesized existing evidence on whether NBIs appear to be effective in improving mental health outcomes in adults.

## METHOD

### Design

The review was conducted in accordance with the PRISMA guidelines (Page et al., 2021) and the Cochrane Handbook (Higgins et al., 2019) and has been preregistered in PROSPERO (CRD42025630035). The eligibility criteria, search strategy, and selection process were developed based on the PICOS framework (Patient/Population; Intervention; Comparison, Outcome; Study design).

### Inclusion criteria

#### Population

Adults aged 18 years and older.

#### Intervention

Included were interventions conducted either individually or in groups, taking place in natural outdoor environments or indoors with a focus on nature exposure.

Such as gardening (e.g., indoors or outdoors tending and cultivating a garden), green exercise (e.g., outdoor physical activities with focus on nature exposure), nature walking (e.g., walking outside in a nature setting), horticultural therapy (e.g., use of plants and plant‐related activities), and forest bathing (e.g., spending time mindfully in natural surroundings).

Interventions that do not involve real nature (e.g., virtual reality) were excluded.

#### Comparators

Studies comparing different types of NBIs were excluded. Only studies comparing NBI with non‐NBI conditions were considered. Acceptable control conditions included placebo conditions (e.g., placebo medication, sham therapy, active control groups), treatment as usual (TAU), and waiting list control groups. Comparisons typically involved urban versus nature‐based activities, or conventional therapy versus nature‐based therapy.

#### Outcome

Included were measures of mental health such as anxiety, depression, loneliness, stress, affect, mood, well‐being, and burnout. Although burnout was not specified in the preregistered protocol or included as a specific search term in the initial search strategy, it was reported in several included studies and was therefore added as a relevant outcome due to its significance to mental health. The health‐related outcomes needed to be assessed in at least one of the primary or secondary outcomes. Standardized instruments, such as self‐reported or external rated scales and questionnaires, were used for outcome assessment. In line with the preregistered protocol, the review focused on psychological mental health outcomes; objectively measured physiological outcomes related to mental health, such as neuroimaging data, cortisol levels, or heart rate variability, were outside the planned scope and were therefore not analyzed.

#### Study design

Only RCTs were included.

### Information sources and search strategy

The systematic search process began in November 2024, and screening of identified records continued until May 2025. The final database search was conducted on December 10, 2024, across the following databases: PubMed, Cochrane Library (Trials), Web of Science, and PsycINFO. ClinicalTrials.gov was also searched for ongoing studies. The aim was to identify as many relevant and currently available studies as possible. In addition to the database search, the reference lists of all included studies were screened in accordance with the preregistered protocol. This citation screening was performed on August 1, 2025.

The following search terms were used to capture a broad range of relevant studies: (Nature based intervention OR gardening OR green exercise OR ecotherapy OR nature based walking OR forest bathing OR horticulture OR community farming OR nature based arts OR environmental conservation) AND (mental health OR mental disorder OR depression OR anxiety OR stress OR affect OR mental well being OR loneliness OR negative mood) AND (RCT OR randomized controlled trials). For the full list of search terms, see Data S1.

### Data selection and collection process

The records retrieved from the databases were exported as CSV files and imported into Rayyan software (Ouzzani et al., 2016) for study management and screening. Titles and abstracts were screened in duplicate and independently by three reviewers (N.Z., C.L.H., and D.H.). For the full‐text screening, an additional reviewer (E.H.) was involved, and all full texts were likewise screened in duplicate. Duplicate records were removed, and all screenings were conducted independently. Upon completion, the reviewers compared their decisions, and any discrepancies were resolved through discussion. As consensus was reached, no further arbitration was required. The study selection process is illustrated in a PRISMA 2020 flow diagram (Page et al., 2021).

### Data extraction

Data were extracted independently and in duplicate by N.Z. and E.H. and subsequently reviewed and verified by D.H. Any discrepancies were resolved through discussion, with the involvement of another reviewer if necessary. In the case of missing data, the variable was labeled as “not applicable” (n.a.). The extracted data were recorded in two Excel spreadsheets—one for the study characteristics (Table 1) and one for the results (Table 2)—and included the following variables: in Table 1 first author, publication year, sample size, mean age and standard deviation, gender, intervention group (including type, duration, frequency, and format), control condition type, trial design (superiority, non‐inferiority) and in Table 2 first author, publication year, intervention and control conditions, outcome measure, mean and standard deviation for pre‐ and post‐measures, type of comparison, *p* values, effect size, effect of NBI over control condition, follow‐up mean and SD, and *p* value for follow‐up.

**TABLE 1:** List of included studies and their characteristics.

Study	Country	Sample size		Sample type	Mean age (SD)		Gender (% female)	Intervention				Control		Trial design
		Intervention	Control		Intervention	Control		Type	Duration	Frequency	Format	Type	Other conditions	
Ameli et al. (2021)	USA	12	12	Volunteers in a military facility	35 (n.a.)^a^		75	Nature walk	1 week	1/week 20 min	Individual	Urban walk	Same	Superiority, crossover
de Bloom et al. (2017)	Finland	Spring: 28 Fall: 23	Spring: C1: 23 C2: 32 Fall: C1: 23 C2: 24	Workers	Spring: 48.9 Fall: 45.5		Spring: 89.2 Fall: 90.0	Nature walk in park	2 weeks	15 min/day	Individual	C1: Relaxation exercise C2: Usual break (no intervention)	Same	Superiority for C2
Bratman et al. (2015)	USA	30	30	Healthy adults from San Francisco bay area	22.8	22.9	55	Nature walk in park	1 day	1 × 50 min	Individual	Urban walk	Same	Superiority
Brito et al. (2024)	Portugal	51	53	Healthy adults under the age of 50	25.2 (5.1)	24.8 (7.0)	32	Green exercise (calisthenics in park)	1 day	1 × 13 min	Group	Calisthenics indoor	Same	Superiority
Brown et al. (2014)	UK	32	C1: 33 C2: 29	Office workers	42 (10.6)^a^		21	Nature walk	8 weeks	2/week a 20 min	Group	C1: Build walking C2: Waitlist	Same for C1 and C2	Superiority
Calogiuri et al. (2015)	Norway	7	7	Municipality employees	49 (8.0)^a^		50	Green exercise	3 days	2 × 45 min	Group	Indoor exercise	Same	Superiority
Chu et al. (2019)	Taiwan	75	75	Older residents of nursing homes	79 (n.a.)^a^		63	Horticulture therapy	8 weeks	1/week 2 h	Group	Routine leisure activities	n.a.	Superiority
Daniels et al. (2022)	Belgium	25	24	Employees of the province of Limburg	45.4 (7.3)	43.7 (10.6)	73	Nature walk	3 weeks	2/week 1.5–2 h	Group	No intervention	n.a.	Superiority
Djernis et al. (2021)	Denmark	22	C1: 20 C2: 18	University students with moderate to high stress levels	31.3 (9.6)	C1: 31.7 (7.5) C2: 28.6 (5.9)	87	MBSR in therapy garden	5 days	All days	Group	C1: MBSR indoor C2: No intervention	C1: Same	Superiority
Gelkopf et al. (2013)	Israel	22	20	Chronic combat‐related PTSD veterans	39.1 (12.4)	37.5 (13.6)	0	Green exercise (sailing)	1 year	1/week 30 min	Group	Waitlist	n.a.	Superiority
Grafetstätter et al. (2017)	Austria	I1: 33 I2: 32	C: 26	Persons working in care professions with moderate to high stress levels	33.9 (10.4)^a^		52	I1: Nature contact (waterfall) + water aerosol contact I2: Nature contact (waterfall)	1 week	Daily 1 h	Group	No intervention	I1 and I2: Vaccine on days 0 and 6	Superiority
Grazuleviciene et al. (2016)	Lithuania	10	10	Stable CAD patients	62.3 (12.6)^a^		35	Nature walk	1 week	Daily 30 min	Group	Urban walk	Same	Superiority
Ho et al. (2022)	China	48	42	University employees	34.8 (9.3)^a^		89	Nature contact	10 days	Daily 30 min	Group	Usual lunch break	Same	Superiority
Huber et al. (2019)	Austria	I1: 27 I2: 26	C2: 27	Persons diagnosed with nscLBP	52.9 (6.4)	C1: 53.3 (8.3) C2: 43.8 (12.1)	56	I1: Green exercise (hiking) I2: Green exercise + balneotherapy	8 days	Daily 5 h	Group	No intervention	n.a.	Superiority
Hyvönen et al. (2023)	Finland	59	77	Persons diagnosed with depression	45.2 (n.a.)		82	Group meetings in park + TAU	12 weeks	1/week 90 min	Group	TAU (treatment contact in a health care service)	n.a.	Superiority
Joubert et al. (2024)	France	106	105	Adult psychiatric inpatients	41.1 (n.a.)	44.4 (n.a.)	50	Horticulture therapy	4 weeks	2/week 90 min	Group	TAU (standard psychiatric care)	n.a.	Superiority
Kam and Siu (2010)	China	12	12	Persons with psychiatric illness	44.3 (11.6)^a^		29	Horticulture therapy	2 weeks	10 × 1 h	Group	Conventional workshop training	n.a.	Superiority
Kavanaugh et al. (2022)	Switzerland	34	22	Physicians and other healthcare workers	39.8 (n.a.)^a^		82	Forest bathing	1 day	1 × 3 h	Group	Waitlist	n.a.	Superiority
Keenan et al. (2021)	UK	25	25	Persons living with depression or anxiety	40.3 (12.7)^a^		60	Nature walk	5 days	Daily 30 min	Group	Urban walk	Same	Superiority
Kim and Park (2018)	Korea	18	18	Married middle‐aged women	40–59^b^		100	Horticulture therapy	6 weeks	2/week 1 h	Group	No intervention	n.a.	Superiority
Koselka et al. (2019)	USA	38	38	Physically healthy young adults	22.9 (4.6)^a^		53	Nature walk	4 weeks	3 × 50 min	Individual	Urban walk	Same	Superiority, crossover
Lai et al. (2018)	China	46	50	Nursing home residents over the age of 70	84.6 (7.2)^a^		66	Horticulture therapy	8 weeks	1/week 1 h	Group	Social activities	n.a.	Superiority
Legrand et al. (2022)	France	50	C1: 50 C2: 50	University students from the Sport and Physical Education Department	20.2 (1.3)^a^		69	Nature walk	1 day	1 × 30 min	Group	C1: Urban walk C2: Regular class	C1: Same C2: 1h	Superiority
Legrand et al. (2025)	France	22	C1: 22 C2: 22	University students	20.9 (0.8)	C1: 20.9 (0.9) C2: 20.4 (0.8)	36	Green exercise (group running)	5 weeks	1/week 1 h	Group	C1: Urban running C2: Watch YouTube	Same for C1 and C2	Superiority
Litt et al. (2023)	USA	145	146	Community residents	41.5 (13.5)^a^		82	Community gardening	6 weeks	n.a.	Group	Waitlist	n.a.	Superiority
Lyu et al. (2019)	China	45	15	Healthy male university students	21.8 (0.3)	21.6 (0.3)	0	Forest bathing	3 days	Daily 8 h	Group	City bathing	Same	Superiority
Ma et al. (2023)	UK	52	52	University students with sleep problems	23.6 (2.2)^a^		90	Nature walk	1 week	Daily 30–35 min	Individual	Urban walk	Same	Superiority
Makizako et al. (2019)	Japan	30	C1: 30 C2: 29	Community‐dwelling older adults with depressive symptoms and mild memory decline	73.1 (5.5)^a^		51	Horticulture therapy	20 weeks	1/week 60–90 min	Group	C1: Exercise C2: Educational program	Same	Superiority
Mao, Cao, et al. (2012)	China	12	12	Patients with diagnosed essential hypertension	60–75^b^		n.a.	Nature walk	1 week	2× daily 90 min	Group	Urban walk	Same	Superiority
Mao, Lan, et al. (2012)	China	10	10	Healthy male university students	20.8 (0.5)^a^		0	Nature walk	2 days	2× daily 90 min	Group	Urban walk	Same	Superiority
Mao et al. (2017)	China	24	12	Patients with chronic heart failure	71.8 (4.7)^a^		39	Nature walk	4 days	2× daily 90 min	Group	Urban walk	Same	Superiority
Mavrantza et al. (2023)	Norway	30	30	Healthy young adults	24.2 (4.2)^a^		57	Green exercise	1 day	1 × 6 min	Individual	Indoor exercise	Same	Superiority, crossover
Minagar et al. (2023)	Iran	51	51	Persons with mild to moderate depression symptoms	37.6 (9.0)	35.9 (12.5)	78	Horticulture therapy	6 weeks	1/week 3 h	Group	Educational program	Same	Superiority
Ng et al. (2018)	Singapore	29	30	Older adults with no severe medical or psychiatric disorders	67.1 (4.3)^a^		78	Horticulture therapy	24 weeks	1/week and 1/month, respectively, for 3 months 1 h	Group	Waitlist	n.a.	Superiority
Odeh et al. (2022)	Saudi‐ Arabia	15	17	Premenopausal women under the age of 50 without any chronic conditions	32.1 (5.1)	32.8 (5.6)	100	Group gardening	8 weeks	2/week 1 h	Group	Art therapy	Same	Non‐inferiority
Olafsdottir et al. (2020)	Iceland	30	C1: 30 C2: 30	Students of local universities	24.39 (2.61)		69	Nature walk	1 day in 2 periods (no exams/exam period)	40 min	Individual	C1: Walking on a treadmill C2: Nature video‐recording	Same for C1	Superiority
Pálsdóttir et al. (2020)	Sweden	51	50	Patients in the chronic and subacute phase after stroke	67 (n.a.)^a^		60	Horticulture therapy	10 weeks	2/week 3.5 h	Group	TAU (individualized standard care after stroke)	Same	Non‐inferiority
Song et al. (2019)	Japan	12	12	Healthy female university students	21.0 (1.3)^a^		100	Nature walk	1 day	1 × 15 min	Individual	Urban walk	Same	Superiority, crossover
Souter‐Brown et al. (2021)	New Zealand	57	C1: 51 C2: 56	University students and employees	33.1 (12.0)^a^		78	Sensory garden	4 weeks	1/week 30 min	Individual	C1: Urban plaza C2: No intervention	Same for C1	Superiority
South et al. (2018)	USA	117	C1: 107 C2: 118	Community‐dwelling adults	44.6 (15.1)^a^		60	Greening contact	8 weeks	Daily	Group	C1: Trash clean up C2: No intervention	n.a.	Superiority
Stigsdotter et al. (2018)	Denmark	43	41	Work‐incapable persons with adjustment disorder/stress reaction	47.9 (7.8)	44.9 (8.8)	85	NNBT	5 weeks	3/week 3 h	Individual/group	CBT	Same	Non‐inferiority
Triguero‐Mas et al. (2017)	France	26	26	Persons with indications of psychological distress	44.3 (26.2)^a^		58	I1: Green environment I2: Blue environment	12 weeks	1 × 30 min and 1 × 180 min	Individual/group	Urban environment	Same	Superiority, crossover
Tsunetsugu et al. (2007)	Japan	12	12	Male university students	22.0 (1.0)^a^		0	Forest bathing	1 day	15‐min walking, 15‐min watching scenery	Individual	Urban environment	Same	Superiority, crossover
Van Den Berg and Custers (2011)	Netherlands	14	16	Healthy allotment garden owner	57.6 (n.a.)^a^		73	Stressful task, then gardening	1 day	1 × 90 min	Individual	Stressful task, then reading nature‐magazines	Same	Superiority
Vujcic et al. (2017)	Serbia	16	14	Psychiatric patients of a day hospital	45.4 (10.2)^a^		70	Horticulture therapy	4 weeks	3/week 1 h	Group	Occupational + art therapy	Same	Non‐inferiority
Wang et al. (2021)	Brazil	40	40	Young obese adults	19.1 (0.7)^a^		59	Nature walk	1 day	1 × 1.6 km	Group	Indoor walk	Same	Superiority
Yen and Huang (2024)	Taiwan	32	C1: 30 C2: 30	Healthy adults under the age of 50	31.3 (7.3)	C1: 23.8 (2.2) C2: 32.6 (8.7)	62	Nature contact	12 weeks	1/week 30 min	Individual	C1: VR nature contact C2: No intervention	n.a.	Superiority

Abbreviations: ADL, activities of daily living; C, control group; CAD, coronary artery disease; CBT, cognitive behavioral therapy; I, intervention group; MBSR, Mindfulness‐based Stress Reduction; NNBT, Nacadia® nature‐based therapy; nscLBP, nonspecific chronic low back pain; PDSD, posttraumatic stress disorder; TAU, treatment as usual; VR, virtual reality.

^a^ Reported mean age refers to the total sample; separate values for intervention and control groups were not provided in the original publication.

^b^ Reported range refers to the total sample; separate mean age for intervention and control groups were not provided in the original publication.

**TABLE 2:** List of included studies and their results.

Study	Intervention	Control	Outcome category	Outcome measure	Post mean (SD)		Type of comparison		Type of comparison	*p* values	Effect size [95% CI]	Effect of NBI over CC	Follow‐up Mean (SD)		Follow‐up *p* values
					Intervention	Control	Intervention	Control					Intervention	Control	
Ameli et al. (2021)	Nature walk	Urban walk	Stress/distress	Stress/distress: DT (Roth et al., 1998)	1.5 (1.0, 2.0)^a^	1.0 (1.0, 2.0)^a^	0.0 (0.0, 0.0)^a^	1.0 (0.0, 4.0)^a^	Between‐group comparison: Post‐intervention Pre–post comparison within groups	Between‐group comparison at post‐intervention: *p* = .02* Pre–post comparison: I: *p* < .01** C: *p* = .34	Between‐group comparison at post‐intervention: *r* = .51 [n.a.] Pre–post comparison within groups: I: *r* = .61 [n.a.] C: *r* = .19 [n.a.]	Yes	n.a.	n.a.	n.a.
de Bloom et al. (2017)	Nature walk in park	C1: Relaxation exercise C2: Usual break (no intervention)	Psychological well‐being	Well‐being: Restoration, fatigue (Demerouti et al., 2012; Ryan & Frederick, 1997; Van Hoof et al., 2007)	Spring RCT n.s., therefore not shown Fall RCT Fatigue: Lunch: 3.35 (1.5) Afternoon: 4.04 (1.5) Evening: 5.41 (1.2) Restoration: Lunch: 4.50 (1.0) Afternoon: n.a. Evening: 4.78 (1.2)	Spring RCT n.s., therefore not shown Fall RCT Fatigue: Lunch: C1: 3.39 (1.3) C2: 3.09 (1.4) Afternoon: C1: 3.78 (1.4) C2: 3.80 (1.5) Evening: C1: 5.07 (1.3) C2: 5.02 (1.3) Restoration: Lunch: C1: 4.20 (1.0) C2: 4.93 (0.9) Afternoon: C1: n.a. C2: n.a. Evening: C1: 5.15 (1.3) C2: 4.66 (1.1)	Spring RCT n.s., therefore not shown Fall RCT During intervention: Fatigue: Lunch: 2.28 (1.0) Afternoon: 3.33 (1.2) Evening: 5.29 (0.9) Restoration: Lunch: 4.93 (0.8) Afternoon: n.a. Evening: 5.11 (0.9) Post intervention: Fatigue: Lunch: 3.10 (1.5) Afternoon: 3.55 (1.7) Evening: 5.18 (1.2) Restoration: Lunch: 4.36 (1.0) Afternoon: n.a. Evening: 4.93 (1.3)	Spring RCT n.s., therefore not shown Fall RCT During intervention: Fatigue: Lunch: C1: 3.03 (0.8) C2: 3.41 (1.1) Afternoon: C1: 3.29 (1.0) C2: 3.94 (1.4) Evening: C1: 4.95 (1.0) C2: 5.24 (1.3) Restoration: Luch: C1: 4.85 (0.8) C2: 4.9 (1.0) Afternoon: C1: n.a. C2: n.a. Evening: C1: 5.03 (1.1.) C2: 4.86 (1.3) Post‐intervention: Fatigue: Lunch: C1: 3.4 (1.6) C2: 2.95 (1.3) Afternoon: C1: 4.26 (1.3) C2: 4.34 (1.8) Evening: C1: 5.11 (1.4) C2: 5.11 (1.7) Restoration: Lunch: C1: 4.52 (1.2) C2: 5.07 (1.2) Afternoon: C1: n.a. C2: n.a. Evening: C1: 4.71 (1.3) C2: 4.72 (1.5)	Group × time interaction Repeated measures ANOVA	Fall RCT Group × time interaction: I vs. C1 and C2: Afternoon: *p* < .05* (fatigue) Lunch/evening: n.s. (fatigue) Lunch/afternoon/evening: n.s. (restoration) Spring RCT n.s.	Fall RCT Comparison within groups during the intervention: I: *d* = .54 (fatigue) Pre–post comparison within groups: I: *d* = .29 (fatigue)	Partial	n.a.	n.a.	n.a.
Bratman et al. (2015)	Nature walk	Urban walk	Anxiety Affective states	Anxiety: STAI (Spielberger et al., 1970) Affective state: PANAS (PA, NA) (Watson et al., 1988) Rumination: RRQ (Trapnell & Campbell, 1999)	n.a	n.a.	n.a.	n.a.	Group × time interaction: 2 × 2 design, mixed factorial ANOVA	Group × time interaction: *p* < .05* (STAI) *p* < .05* (RRQ) *p* < .001*** (PA) *p* < .05* (NA)	n.a	Yes	n.a.	n.a.	n.a.
Brito et al. (2024)	Green exercise (calisthenics in park)	Calisthenics indoor	Affective states	Affective state: FS (Hardy & Rejeski, 1989)	3 (1.5)	2.8 (1.8)	2.6 (1.9)	2.5 (1.5)	Group × time intercation: 2 × 2 design, MANOVA	Group × time interaction: *p* = .54	n.a.	No	n.a.	n.a.	n.a.
Brown et al. (2014)	Nature walk in park	C1: Built walking C2: Waitlist	Psychological well‐being	Well‐being: SF‐8 (Yiengprugsawan et al., 2023)	50.3 (6.3)	C1: 50.3 (9.7) C2: 50.7 (9.2)	53 (6.1)	C1: 50.1 (9.8) C2: 47.4 (8.2)	Group × time interaction: Mixed design ANOVA	Group × time interaction: I vs. C1 and C2: *p* < .05* (no post hoc analysis)	n.a.	Yes	n.a.	n.a.	n.a.
Calogiuri et al. (2015)	Green exercise	Indoor exercise	Affective states	Affective state: PAAS (Lox et al., 2000)	n.a.	n.a.	3.5 (0.1)^b^	2.8 (0.1)^b^	Between‐group comparison at post‐intervention (adjusted for (1) baseline and (2) pre‐exercise values)	Between‐group comparison at post‐intervention: *p* = .001*** (adjusted for baseline values) *p* = .06 (adjusted for pre‐exercise values)	n.a.	Partial	10 weeks: 3.2 (0.1)^b^	10 weeks: 2.8 (0.1)^b^	10 weeks: Between‐group comparison at follow‐up (adjusted for baseline values): *p* = .02*
Chu et al. (2019)	Horticulture therapy	Routine leisure activities	Depression Other outcomes	Depression: GDS‐15 (Yesavage & Sheikh, 1986) Loneliness: UCLA (Russell et al., 1978)	7.3 (0.4) 50.8 (0.7)	5.4 (0.4) 49.6 (0.9)	2.7 (0.3) 44.3 (0.5)	8.1 (0.4) 52.6 (0.6)	Group × time interaction; GEE Pre–post comparison within groups	Group × time interaction: *p* < .001*** (GDS‐15) *p* < .001*** (UCLA) Pre–post comparison: *p* < .001*** (GDS‐15) *p* < .001*** (UCLA)	n.a.	Yes	n.a.	n.a.	n.a.
Daniels et al. (2022)	Nature walk	No intervention	Burnout	Burnout: BAT (De Beer et al., 2020)	58.1 (2.2)^c^	56.4 (2.1)^c^	54.5 (2.1)^c^	57.2 (2.2)^c^	Group × time interaction; GEE Pre–post comparison within groups	Group × time interaction: *p* < .001*** Pre–post comparison: I: *p* < .05* C: *p* > .05	n.a.	Yes	2 weeks: 51.9 (2.1) 3 weeks: 48.5 (2.1)	2 weeks: 57.4 (2.2) 3 weeks: 57.2 (2.2)	Pre‐follow‐up comparison: 2 weeks: I: *p* < .05* C: *p* > .05 3 weeks: I: *p* < .05* C: *p* > .05
Djernis et al. (2021)	MBSR in therapy garden	C1: MBSR indoor C2: No intervention	Other outcomes Stress/distress	Self‐compassion: SCS‐SF (Neff, 2012) Stress/distress: PSS‐10 (Cohen et al., 1983)	34.0 (7.4) 21.6 (4.8)	C1: 34.1 (6.4) 21.8 (6.5) C2: 34 (9.3) 21.8 (5.3)	39.8 (7.8) 31.2 (3.6)	C1: 36.7 (10.5) 19.4 (7.7) C2: 33.8 (10.7) 22.9 (6.7)	Group × time interaction; LMM	Group × time interaction: *p* > .05 (SCS‐SF) *p* > .05 (PSS‐10)	Group × time interaction: I vs. C1: *g* = .03 [n.a.] (SCS‐FS) I vs. C2: *g* = .37 [n.a.] (SCS‐SF) I vs. C1: *g* = .36 [n.a.] (PSS‐10) I vs. C2: *g* = .34 [n.a.] (PSS‐10)	No	3 months: 39.8 (7.8) (SCS‐SF) 31.2 (3.6) (PSS‐10)	3 months: C1: 41.1 (6.9) (SCS‐SF) 30.1 (3.8) (PSS‐10) C2: 35.5 (8.9) (SCS‐SF) 33.5 (3.5) (PSS‐10)	3 months: Group × time interaction: C1: *p* = .752 (SCS‐SF) C2: *p* = .030* (SCS‐SF) C1: *p* = .578 (PSS‐10) C2: *p* = .276 (PSS‐10) Pre–post comparison: I: *p* < .05* (SCS)
Gelkopf et al. (2013)	Green exercise (sailing)	Waitlist	Depression Other outcomes	Depression: BDI‐FS (Kliem et al., 2014) Posttraumatic stress: SASRQ (Cardeña et al., 2000)	15.5 (2.7) 115.1 (16.0)	15.1 (4.2) 111.3 (26.6)	14.0 (2.9) 105.4 (21.5)	15.6 (3.8) 113.6 (19.2)	Group × time interaction; MANOVA	Group × time interaction: *p* = .04* (BDI‐FS) *p* = .04* (SASRQ)	Group × time interaction: *η* ^*2*^ = .11 [n.a.] (BDI‐FS) *η* ^*2*^ = .11 [n.a.] (SASRQ)	Yes	n.a.	n.a.	n.a.
Grafetstätter et al. (2017)	I1: Nature contact (waterfall) + water aerosol contact I2: Nature contact (waterfall)	C: No intervention	Depression Anxiety Mood Stress/distress Burnout	Depression: SCL‐90‐D Anxiety: SCL‐90‐A (Klaghofer & Brähler, 2002) Mood: Bf‐S Stress recovery: EBF (Kallus, 1995) Burnout: MBI‐D (Büssing & Perrar, 1992)	n.a.	n.a.	n.a.	n.a.	Group × time interaction; LMM	Group × time interaction: I1 vs. C: *p* = .006** (SCL‐90‐D) *p* = .005** (SCL‐90‐A) *p* = .05 (bf‐S) *p* = .07 (EBF) *p* = .064 (MBI‐D‐depersonalization) *p* > .05 (MBI‐D‐emotional exhaustion; personal accomplishment) I2 vs. C: *p* = .036* (SCL‐90‐D) *p* = .042* (SCL‐90‐A) *p* = .06 (bf‐S) *p* = .07 (EB F) *p* = n.a. (MBI‐D)	n.a.	Partial	66 days: n.a.	66 days: n.a.	66 days: Group × time interaction: I1 vs. C: n.a. (SCL‐90‐D/A) n.a. (bf‐S) n.a. (EBF) *p* = .002** (MBI‐D‐depersonalization) *p* = n.s. (MBI‐D‐emotional exhaustion; personal accomplishment) Pre‐follow‐up comparison: I1: *p* = n.s. (SCL‐90‐D) *p* = n.s. (SCL‐90‐A) n.a. (bf‐S) *p* = .036* (EBF) n.a (MBI‐D‐depersonalization) n.a (MBI‐D‐emotional exhaustion; personal accomplishment)
Grazuleviciene et al. (2016)	Nature walk in park	Urban walk	Affective states	Affective state: PANAS (PA, NA)	PA: 26.2 (1.8)^c^ NA: 13.1 (0.8)^c^	PA: 26.0 (1.1)^c^ NA: 15.9 (1.9)^c^	PA: 0.5^d^ NA: 0^d^	PA: −1.50^d^ NA: −1.50^d^	Pre–post comparison within groups	Pre–post comparison: I: *p* = .322 (PA) *p* = .453 (NA) C: *p* = .422 (PA) *p* = .297 (NA)	n.a.	n.a.	n.a.	n.a.	n.a.
Ho et al. (2022)	Nature contact in garden	Usual lunch break	Anxiety Depression Stress/distress Psychological well‐being Affective states Burnout	Anxiety: GAD‐7 Depression: PHQ‐9 Stress/distress: PSS‐10 Well‐being: WHO‐5 (Sischka et al., 2020), SWLS (Diener at al., 1985) Affective state: CAS‐PA (Hamid & Cheng, 1996) Burnout: MBI‐GS (Maslach & Jackson, 1981)	GAD‐7: 5.5 (3.2) PHQ‐9: 5.6 (3.3) PSS‐10: 29.4 (4.4) WHO‐5: 13.0 (4.2) SWLS: 12.2 (1.9) CAS‐PA: 10.9(3.0) MBI‐GS: 31.6 (6.8)	GAD‐7: 7.0 (5.4) PHQ‐9: 6.9 (4.6) PSS‐10: 30.4 (5.2) WHO‐5: 13.1 (5.6) SWLS: 12.2 (1.9) CAS‐PA: 11.6 (4.1) MBI‐GS: 33.5 (9.9)	n.a.	n.a.	Group × time interaction; ANOVA Between‐group comparison of change scores	Group × time interaction: *p* = .010* (GAD‐7) *p* = .002** (PHQ‐9) *p* = .001** (PSS‐10) *p* = .005** (WHO‐5) *p* = .354 (SWLS) *p* = .083 (CAS‐PA) *p* = .100 (MBI‐GS) Between‐group comparison of change scores: *p* < .01** (GAD‐7) *p* < .01** (PHQ‐9) *p* < .001*** (PSS‐10) *p* < .001*** (WHO‐5) *p* < .01** (SWLS) *p* < .001*** (CAS‐PA) *p* < .05* (MBI‐GS)	n.a.	Partial	3 months: n.a.	3 months: n.a.	3 months: Group × time interaction: *p* = .751 (GAD‐7) *p* = .162 (PHQ‐9) *p* = .019* (PSS‐10) *p* = .899 (WHO‐5) *p* = .314 (SWLS) *p* = .454 (CAS‐PA) *p* = .272 (MBI‐GS)
Huber et al. (2019)	I1: Green exercise (hiking) I2: Green exercise + balneotherapy	No intervention	Psychological well‐being	Well‐being: WHO‐5	I1: 16 (4.9) I2: 13.2 (6.0)	14.5 (5.2)	I1: 18 (5.3) I2: 16.5 (5.4)	14.4 (5.4)	Group × time interaction; LMM	Group × time interaction: I1: *p* = .057 I2: *p* < .001***	n.a.	Partial	4 months: n.a.	4 months: n.a.	4 months: Group × time interaction: All *p* > .05
Hyvönen et al. (2023)	Group meetings in park + TAU (treatment contact in a healthcare service)	TAU (treatment contact in a healthcare service)	Depression Stress/distress	Depression: BDI‐I Stress/distress: CORE‐10 (Barkham et al., 2013)	24.6 (10.7) 19.4 (6.8)	23.8 (9.1) 18.6 (6.7)	20.9 (11.1) 15.6 (7.3)	21.9 (10.1) 18.1 (7.0)	Group × time interaction; LMM Pre–post comparison within groups	Group × time interaction (includes pre/post/follow‐up): *p* = n.s. (BDI‐I) *p* < .05* (CORE‐10) Time effect (includes pre/post/follow‐up): I: *p* < .001***( BDI‐I) C: *p* < .05* (BDI‐I) I: *p* < .001*** (CORE‐10) C: *p* = n.s. (CORE‐10)	Between group comparison of change scores: *d* = .16 (BDI) *d* = .42 (CORE‐10)	Partial	3 months: 19.2 (12.0) (BDI‐I) 15.2 (7.2) (CORE‐10)	3 months: 21.5 (10.3) (BDI‐I) 17.3 (7.7) (CORE‐10)	3 months: Group × time interaction (includes pre/post/follow‐up): *p* = n.s. (BDI‐I) *p* < .05* (CORE‐10) Pre‐follow‐up comparison: *d* = .28 (BDI‐I) *d* = .40 (CORE‐10)
Joubert et al. (2024)	Horticulture therapy	TAU (standard psychiatric care)	Anxiety	Anxiety: HADS‐A (Zigmond & Snaith, 1983)	13.7 (2.9)	12.7 (2.9)	9.6 (4.1)	11.0 (4.4)	Between‐group comparison at post‐intervention	Between‐group comparison at post‐intervention: *p* = .017*	n.a.	Yes	8 weeks: 9.6 (4.6)	8 weeks: 10.6 (3.9)	n.a.
Kam and Siu (2010)	Horticulture therapy	Conventional workshop training	Depression Anxiety Stress/distress Psychological well‐being	Depression, anxiety, stress: DASS‐21 (Henry & Crawford, 2005) Well‐being: PWI‐C (Lau et al., 2005)	21.8 (11.9) 49.5 (11.8)	16.1 (14.2) 53.2 (14.9)	−24.2 (17.8)^e^ 0.6 (14.2)^e^	−0.5 (6.8)^e^ 1.5 (6.1)^e^	Between‐group comparison of change scores	Between‐group comparison of change scores: *p* = .01* (DASS‐21) *p* = .04* (DASS‐21, depression subscale) *p* = .01* (DASS‐21, anxiety subscale) *p* = .05 (DASS‐21, stress subscale) *p* = .84 (PWI‐C)	n.a.	Partial	n.a.	n.a.	n.a.
Kavanaugh et al. (2022)	Forest bathing	Waitlist	Burnout	Burnout: OLBI (Oana Tipa et al., 2019) Mini‐Z (Shaholli et al., 2024)	39.0 (n.a.)^e^ 35.0 (n.a.)^e^	42.0 (n.a.)^e^ 36.0 (n.a.)^e^	38.0 (n.a.)^e^ 34.0 (n.a.)^e^	41.0 (n.a.)^e^ 37.0 (n.a.)^e^	Group comparison of post‐intervention scores; *t*‐test Pre–post comparison within groups	Group comparison of post‐intervention scores: *p* = .17 (OLBI) *p* = .03* (Mini‐Z); adj. *p* value n.s. Pre–post comparison: I: *p* = .30 (OLBI) C: *p* = .44 (OLBI) I: *p* = .23 (Mini‐Z) C: *p* = .31 (Mini‐Z)	n.a.	No	n.a.	n.a.	n.a.
Keenan et al. (2021)	Nature walk	Urban walk	Psychological well‐being Affective states	Well‐being: WEMWBS (Tennant et al., 2007) Affective state: PANAS (PA, NA)	WEMWBS: 44.8 (11.8) PA: 15.7 (2.2) NA: 42.6 (4.4)	WEMWBS: 44.0 (12.5) PA: 15.1 (2.1) NA: 43.8 (4.5)	WEMWBS: 53.3 (9.4) PA: 30.4 (4.0) NA: 29.7 (5.7)	WEMWBS: 49.8 (10.7) PA: 17.9 (4.3) NA: 40.2 (6.0)	Group × time interaction; ANOVA (time × condition)	Group × time interaction (includes pre/post/follow‐up): *p* < .001*** (WEMWBS) *p* < .001*** (PA) *p* < .001*** (NA)	Group × time interaction (includes pre/post/follow‐up): *Ηp* ^*2*^ = .29 [n.a.] (WEMWBS) *Ηp* ^2^ = .42 [n.a.] (PA) *Ηp* ^2^ = .66 [n.a.] (NA)	Yes	6 weeks: 63.4 (3.7) (WEMWBS) 36.9 (2.1) (PA) 36.6 (1.7) (NA)	6 weeks: 42.7 (3.7) (WEMWBS) 28.0 (3.0) (PA) 20.8 (3.0) (NA)	6 weeks: Group × time interaction (includes pre/post/follow‐up): *p* < .001*** (WEMWBS) *p* < .001*** (PA) *p* < .001*** (NA)
Kim and Park (2018)	Horticulture therapy	No intervention	Depression Anxiety	Depression: SDS (Zung, 1965) Anxiety: STAI	44.7 (8.6) 84.1 (19.6)	43.2 (6.6) 85.3 (15.3)	33.4 (5.9) 62.8 (12.9)	43.5 (6.8) 87.5 (15.7)	Pre–post comparison within groups	Pre–post comparison: I: *p* < .001*** (SDS) C: *p* = .90 (SDS) I: *p* < .001*** (STAI) C: *p* = .67 (STAI)	n.a.	n.a.	n.a.	n.a.	n.a.
Koselka et al. (2019)	Nature walk	Urban walk	Affective states Anxiety Stress/distress	Affective state: PANAS (PA, NA) Anxiety: STAI Stress/distress: PSS‐10	PA: 28.3 (n.a.) NA: 13.4 (n.a.) STAI: 34.6 (n.a.) PSS‐10: 14.4 (n.a.)	PA: 28.6 (n.a.) NA: 14.2 (n.a.) STAI: 36.1 (n.a.) PSS‐10: 13.6 (n.a.)	PA: 30.0 (n.a.) NA: 12.0 (n.a.) STAI: 31.6 (n.a.) PSS‐10: 13.0 (n.a.)	PA: 28.1 (n.a.) NA: 13.0 (n.a.) STAI: 34.4 (n.a.) PSS‐10: 13.8 (n.a.)	Between‐group comparison of change scores	Between‐group comparison of change scores: *p* = .07 (PA) *p* = .02* (NA) *p* = .04* (STAI) *p* = .04* (PSS‐10)	n.a.	Partial	n.a.	n.a.	n.a.
Lai et al. (2018)	Horticulture therapy	Social activities	Depression Psychological well‐being	Depression: GDS‐15 Happiness: SHS (Lyubomirsky & Lepper, 1999) Well‐being: PWBI (Cummins et al., 2003)	4.2 (3.3) 17.9 (5.6) 66.9 (14.4)	3.8 (3.7) 20.9 (4.5) 66.9 (16.7)	n.a.	n.a.	Group × time interaction; GEE	Group × time interaction: *p* > .05 (GDS‐15) *p* = .036* (SHS) *p* > .05 (PWBI)	SHS: Group × time interaction: ß = 1.457 [.093, 2.822]	Partial	3 months: n.a.	3 months: n.a.	3 months: Group × time interaction: *p* > .05 (GDS‐15) *p* < .05* (SHS) *p* > .05 (PWBI) SHS: ß = 1.457, SE = 0.696 (95% CI: 0.093–2.822)
Legrand et al. (2022)	Nature walk	C1: Urban walk C2: Regular class	Affective states	Affective state: PANAS (PA, NA)	PA: 29.9 (5.8) NA: 16.5 (5.3)	C1: PA: 31.6 (6.8) NA: 17.2 (6.5) C2: PA: 28.4 (5.9) NA: 14.7 (3.8)	PA: 32.6 (6.2) NA: 12.6 (2.6)	C1: PA: 30.2 (7.2) NA: 13.6 (3.7) C2: PA: 26.0 (6.3) NA: 13.6 (3.3)	Group × time interaction; 3 × 2 mixed‐design, ANOVA Pre–post comparison within groups	Group × time interaction: I vs. C1: *p* < .001*** (PA) *p* < .01** (NA) Pre–post comparison: PA: I: *p* = .002** (increase) C1: *p* = .661 C2: *p* = .009** (decrease) NA: I: *p* < .001*** C1: *p* < .001*** C2: *p* = .995	I vs. C1: *d* = .83 [.42, 1.23] (PA) *d* = −.11 [−.57, .28] (NA)	Yes	n.a.	n.a.	n.a.
Legrand et al. (2025)	Green exercise (group running)	C1: Urban running C2: Watch YouTube	Mood	Mood: POMS‐V POMS‐F	POMS‐V: 14.7 (8.3) POMS‐F: 8.4 (7.5)	C1: POMS‐V: 14.7 (5.5) POMS‐F: 9.1 (7.5) C2: POMS‐V: 15.5 (6.7) POMS‐F: 9.8 (8.5)	POMS‐V: 18.9 (3.8) POMS‐F: 6.0 (5.5)	C1: POMS‐V: 18.9 (6.0) POMS‐F: 5.7 (5.3) C2: POMS‐V: 11.9 (7.2) POMS‐F: 12‐1 (8.2)	Group × time interaction; 3 × 2 design, ANOVA	Group × time interaction (all groups): *p* = .003** (POMS‐V) *p* = .019* (POMS‐F)	Group × time interaction: I vs. C1: n.s. I vs. C2: *d* = .96 [.24, 1.66] (POMS‐V) *d* = −.87 [−1.57, −.16] (POMS‐F)	Partial	n.a.	n.a.	n.a.
Litt et al. (2023)	Community gardening	Waitlist	Stress/distress Anxiety	Stress/distress: PSS‐10 Anxiety: GAD‐7	n.a.	n.a.	n.a.	n.a.	Between‐group comparison of change scores	Between‐group comparison of change scores: *p* = .58 (PSS‐10) *p* = .49 (GAD‐7)	n.a.	No	n.a.	n.a.	n.a.
Lyu et al. (2019)	Forest bathing	City bathing	Mood	Mood: POMS	n.a	n.a	n.a	n.a	Pre–post comparison within groups	Pre–post comparison: I: *p* < .05* (decrease in mood disturbance) C: *p* < .05* (increase in mood disturbance)	n.a.	n.a.	n.a.	n.a.	n.a.
Ma et al. (2023)	Nature walk in park	Urban walk	Mood	Mood: POMS	29.8 (4.3)^h^	29.2 (4.4)^h^	27.3 (4.0)^h^	27.8 (3.7)^h^	Group × time interaction; 2 × 2 design, ANOVA	Group × time interaction: *p* = .44	Group × time interacation: *η* ^2^ < .01	No	5 days: 26.4 (3.2)^h^	5 days: 26.7 (3.9)^h^	5 days: Group × time interaction: *p* = .86
Makizako et al. (2019)	Horticulture therapy	C1: Exercise C2: Educational program	Depression Psychological well‐being	Depression: GDS‐15 Well‐being: SF‐12 (Ware et al., 1996)	GDS‐15: 6.9 (4.7) SF‐12: 50.8 (7.0)	C1: GDS‐15: 7.1 (2.5) SF‐12: 49.5 (7.9) C2: GDS‐15: 6.4 (2.5) SF‐12: 52.4 (6.2)	GDS‐15:4.7 (2.7) 51.9 (7.7)	C1: GDS‐15: 5.3 (2.5) SF‐12: 50.3 (7.1) C2: GDS‐15:5.1 (3.1) SF‐12: 50.1 (7.5)	Between‐group comparison at post intervention controlled for baseline Pre–post comparison within groups	Between‐group comparison at post‐intervention controlled for baseline: *p* = .744 (GDS‐15) *p* > .05 (SF‐12) Pre–post comparison: GDS‐15: I: *p* = .001** (decrease) C1: *p* = .002** (decrease) C2: *p* = .001** (decrease) SF‐12: All groups: *p* > .05	n.a.	No	12 months: 5.0 (3.5) (GDS‐15) 55.0 (6.2) (SF‐12)	12 months: C1: 5.3 (2.9) (GDS‐15) 49.2 (9.4) (SF‐12) C2: 4.1 (3.4) (GDS‐15) 51.5 (5.8) (SF‐12)	12 months: Between‐group comparison at post‐intervention controlled for baseline: *p* = .741 (GDS‐15) *p* > .05 (SF‐12) Pre‐follow‐up comparison: GDS‐15: I: *p* < .05* (decrease) C1: *p* < .05* (decrease) C2: *p* < .05* (decrease) SF‐12: All groups: *p* > .05
Mao, Cao, et al. (2012)	Nature walk	Urban walk	Mood	Mood: POMS	n.a.	n.a.	n.a.	n.a.	Pre–post comparison within groups	Pre–post comparison: I: *p* < .05* (decrease in subscales depression‐dejection, anger‐hostility, fatigue‐inertia, confusion‐bewilderment) C: *p* > .05	n.a.	n.a.	n.a.	n.a.	n.a.
Mao, Lan, et al. (2012)	Nature walk	Urban walk	Mood	Mood: POMS	n.a.	n.a.	n.a.	n.a.	Between‐group comparison at post‐intervention	Between‐group comparison at post‐intervention: *p* < .05* (lower levels of tension‐anxiety, depression‐dejection, anger‐hostility, fatigue‐interia, confusion‐bewilderment and higher levels of vigor activity in I vs. C)	n.a.	Yes	n.a.	n.a.	n.a.
Mao et al. (2017)	Nature walk	Urban walk	Mood	Mood: POMS	n.a.	n.a.	n.a.	n.a.	Between‐group comparison at post‐intervention Pre–post comparison within groups	Between‐group comparison at post‐intervention: *p* < .05* (lower levels of tension‐anxiety and depression‐dejection) Pre–post comparison: I: *p* < .05* (decrease in depression‐dejection, anger‐hostility and confusion‐bewilderment) C: *p* > .05	n.a.	Yes	n.a.	n.a.	n.a.
Mavrantza et al. (2023)	Green exercise	C1: Indoor exercise C2: Virtual green exercise	Affective states	Affective state: FS	n.a.	n.a.	n.a.	n.a.	Group × time interaction; repeated‐measures, ANOVA Within exercise group comparison	Group × time interaction (I vs. C1 and C2): *p* = .138 Within exercise group comparison: *p* = .003** (I vs. C1) *p* = .014* (I vs. C2)	I vs. C1 and C2: Group × time interaction: *η* ^2^ = .063	Partial	n.a.	n.a.	n.a.
Minagar et al. (2023)	Horticulture therapy	Educational program	Depression	Depression: BDI‐II	n.a.	n.a.	n.a.	n.a.	Group × time interaction; mixed‐model, ANOVA^i^	Group × time interaction: Intention‐to‐treat analysis: *p* = .004** Complete‐case analysis: *p* < .001***	n.a.	Yes	1 month: n.a.	1 month: n.a.	1 month: Group × time interaction: Intention‐to‐treat analysis: *p* = .033* Complete‐case analysis: *p* = .016*
Ng et al. (2018)	Horticulture therapy	Waitlist	Depression Anxiety Psychological well‐being	Depression: SDS Anxiety: SAS (Zung, 1971) Well‐being: PWB (Ryff, 1989), SWLS	SDS: 44.7 (3.8) SAS: 35.1 (2.2) PWB: 28.1 (4.9) SWLS: 79.8 (6.3)	SDS: 45.3 (5.3) S34.2 (2.5) PWB: 27.9 (6.6) SWLS: 76.5 (7.6)	n.a	n.a.	Group × time interaction; rmANOVA	Group × time interaction (includes pre/post/follow‐up): *p* = .68 (SDS) *p* = .81 (SAS) *p* = .34 (PWB) *p* = .64 (SWLS)	n.a.	No	6 months: n.a.	6 months: n.a.	Group × time interaction (includes pre/post/follow‐up): *p* = .68 (SDS) *p* = .81 (SAS) *p* = .34 (PWB) *p* = .64 (SWLS)
Odeh et al. (2022)	Group gardening	Art therapy	Mood Depression Anxiety Stress/distress Psychological well‐being	Mood: POMS Depression: BDI‐II Anxiety: STAI Stress/distress: PSS Well‐being: SF‐36 (mental) (Ware et al., 2007)	53.1 (9.0)^h^ 8.2 (6.8) 34.3 (11.1) 14.9 (4.1) 45.5 (10.6)	53.5 (9.6)^h^ 9.0 (6.3) 32.1 (9.3) 15.8 (7.2) 45.5 (11.3)	46.9 (7.4)^h^ 2.8 (3.6) 29.3 (6.6) 9.4 (5.6) 48.6 (6.5)	47.0 (10.0)^h^ 5.1 (6.6) 32.2 (7.8) 10.0 (6.8) 47.3 (8.9)	Group × time interaction; rmANOVA Pre–post comparison within groups	Group × time interaction: *p* = .92 (POMS) *p* = .41 (BDI‐II) *p* = .15 (STAI) *p* = .90 (PSS) *p* = .72 (SF‐36) Pre–post comparison: I: *p* = .018* (POMS) C: *p* = .009** (POMS) I: *p* < .001*** (BDI‐II) C: *p* = .009** (BDI‐II) I: *p* > .05 (STAI) C: *p* > .05 (STAI) I: *p* = .002** (PSS) C: *p* < .001*** (PSS) I: *p* > .05 (SF‐36) C: *p* > .05 (SF‐36)	Pre–post comparison: I: *d* = −.81 [−1.48, −.01] (POMS) C: *d* = −.60 [−1.30, −.07] (POMS) I: *d* = −.90 [−1.46, .02] (BDI‐II) C: *d* = −.74 [−1.46, −.06] (BDI‐II) I: *d* = −.45 [−1.09, .36] (STAI) C: *d* = −.01 [−.66, .68] (STAI) I: *d* = −.97 [−1.94, −.39] (PSS) C: *d* = −.85 [−1.52, −.12] (PSS) I: *d* = .32 [−.45, .99] (SF‐36) C: *d* = .19 [−.51, −.84] (SF‐36)	No (non‐inferiority)	n.a.	n.a.	n.a.
Olafsdottir et al. (2020)	Nature walk	C1: Walking on a treadmill C2: Nature video recording	Affective states	Affective state: PANAS (PA, NA)	No exams: PA: 31.10 (5.97) NA: 13.11 (2.32) Exam period: PA: 31.30 (5.89) NA15.83 (3.94)	No exams: C1: PA: 30.08 (6.53) NA:15.13 (5.35) C2: PA: 32.04 (5.47) 14.61 (3.50) Exam period: C1: PA:29.88 (6.41) NA: 17.21 (5.73) C2: PA: 32.13 (5.49) NA: 18.57 (6.49)	No exams: PA: 36.80 (9.38) NA: 11.17 (2.43) Exam period: PA: 37.50 (8.37) NA: 11.78 (3.30)	No exams: C1: PA: 29.96 (6.50) NA: 12.96 (3.06) C2: PA: 26.04 (7.57) NA: 11.39 (1.80) Exam period: C1: PA: 29.88 (6.42) NA: 14.50 (4.15) C2: PA: 26.04 (7.57) NA: 11.39 (1.80)	3 × 3 × 2 mixed‐design ANOVAs groups as between subject factor, time and period as within‐subject factor, PA, NA as dependent variables	NA: Group × time interaction: n.s. Period: *p* < .001*** (NA higher during exam period) Period × time interaction: *p* < .001*** PA: Group × time interaction: *p* < .001*** (higher PA scores in I compared to C1 and C2) Pre–post comparison: No exams: NA: I: *p* < .001*** C1: *p* < .001*** C2: *p* < .001*** PA: n.a. Exam period: NA: I: *p* < .001*** C1: *p* < .001*** C2: *p* < .001*** PA: n.a.	NA: Group × time interaction: n.s. Period: *η* ^2^ = .19 Period × time interaction: *η* ^2^ = .15 PA: Group × time interaction: *η* ^2^ = .20	Yes	n.a.	n.a.	n.a.
Pálsdóttir et al. (2020)	Horticulture therapy	TAU (individualized standard care after stroke)	Anxiety Depression Burnout	Anxiety: HADS‐A Depression: HADS‐D Burnout: MFS (Johansson et al., 2010)	7.6 (n.a.) 5.4 (n.a.) 11.4 (n.a.)	7.9 (n.a.) 5.9 (n.a.) 12.4 (n.a.)	6.3 (n.a.) 4.3 (n.a.) 8.9 (n.a.)	7.4 (n.a.) 4.7 (n.a.) 11.1 (n.a.)	Between‐group comparison of change scores; Wilcoxon‐rank‐sum test Pre–post comparison within groups	Between‐group comparison of change scores: *p* = .42 (HADS‐A) *p* = .31 (HADS‐D) *p* = .91 (MFS) Pre–post comparison: I: *p* = .005** (HADS‐A) C: *p* = .118 (HADS‐A) I: *p* = .028* (HADS‐D) C: *p* = .003** (HADS‐D) I: *p* = .004** (MFS) C: *p* = .008** (MFS)	n.a.	No (non‐inferiority	14 months: 6.3 (n.a.) (HADS‐A) 4.7 (n.a.) (HADS‐D) 9.7 (n.a.) (MFS)	14 months: 7.2 (n.a.) (HADS‐A) 4.9 (n.a.) (HADS‐D) 11.5 (n.a.) (MFS)	14 months: Between‐group comparison of change scores: *p* = .69 (HADS‐A) *p* = .13 (HADS‐D) *p* = .80 (MFS) Pre‐follow‐up comparison: I: *p* = .054 (HADS‐A) C: *p* = .043* (HADS‐A) I: *p* = .562 (HADS‐D) C: *p* = .029* (HADS‐D) I: *p* = .067 (MFS) C: *p* = .055 (MFS)
Song et al. (2019)	Nature walk	Urban walk	Mood Anxiety	Mood: POMS Anxiety: STAI	n.a.	n.a.	0.1 (4.9)^e,^, ^h^ 34.8 (7.2)^e^	7.7 (7.3)^e,^, ^h^ 45.3 (7.1)^e^	Between‐group comparison at post‐intervention; Wilcoxon signed‐rank test	Between‐group comparison at post‐intervention: *p* < .01** (POMS TMD) *p* < .01** (STAI)	n.a.	Yes	n.a.	n.a.	n.a.
Souter‐Brown et al. (2021)	Sensory garden	C1: Urban plaza C2: No intervention	Psychological well‐being Affective states	Well‐being: Flour. Sc. (Diener et al., 2010) Affective state: SPANE	n.a.	n.a.	n.a.	n.a.	Between‐group comparison of change scores; GLM	Between‐group comparison of change scores: I vs. C1: *p* = .001*** (flour. Sc.) *p* > .05 (SPANE) I vs. C2: All *p* > .05	n.a.	Partial	n.a.	n.a.	n.a.
South et al. (2018)	Greening intervention	C1: Trash cleanup C2: No intervention	Stress/distress	Distress: K6 “nervous” “hopeless” “restless” “depressed” “everything an effort” “worthless” (Kessler et al., 2002)	9.4 (n.a.)^f^ 34.0 (n.a.)^f^ 16.4 (n.a.)^f^ 30.3 (n.a.)^f^ 15.2 (n.a.)^f^ 41.0 (n.a.)^f^ 10.3 (n.a.)^f^	5.5 (n.a.)^f^ 27.9 (n.a.)^f^ 13.2 (n.a.)^f^ 22.8 (n.a.)^f^ 11.8 (n.a.)^f^ 33.8 (n.a.)^f^ 6.6 (n.a.)^f^	3.9 (n.a.)^f^ 23.0 (n.a.)^f^ 8.9 (n.a.)^f^ 17.5 (n.a.)^f^ 10.5 (n.a.)^f^ 31.1 (n.a.)^f^ 5.1 (n.a.)^f^	4.8 (n.a.)^f^ 23.8 (n.a.)^f^ 8.7 (n.a.)^f^ 20.8 (n.a.)^f^ 8.7 (n.a.)^f^ 26.0 (n.a.)^f^ 8.7 (n.a.)^f^	Group × time interaction; difference‐in‐difference analysis	Group × time interaction: I vs. C1: *p* = .49 I vs. C2: *p* = .051 (K6) *p* = .36 (nervous) *p* = .46 (hopeless) *p* = .06 (restless) *p* = .03* (depressed) *p* = .73 (everything an effort) *p* = .04* (worthless)	n.a.	Partial	n.a.	n.a.	n.a.
Stigsdotter et al. (2018)	NNBT	CBT	Psychological well‐being Burnout	Well‐being: PGWBI (Grossi & Compare, 2014) Burnout: SMBQ (Lundgren‐Nilsson et al., 2012)	46.6 (15.4) n.a. (n.a.)	49.2 (16.6) n.a. (n.a.)	61.4 (15.5) n.a. (n.a.)	59.6 (18.9) n.a. (n.a.)	Group × time interaction; mixed‐design, ANOVA (for primary outcome) Between‐group comparison at post‐intervention; Wilcoxon signed rank test Pre–post comparison within groups	Group × time interaction: *p* > .05 (PGWBI) Between‐group comparison at post‐intervention: *p* = .88 (SMBQ) Pre–post comparison: I: *p* < .001*** (PGWBI) C: *p* < .05* (PGWBI) I: *p* < .001*** (SMBQ) C: *p* < .001*** (SMBQ)	PGWBI: Group × time interaction: *η* ^2^ = .01 Effect of NNBT: *η* ^2^ = 0.144, *ω* ^2^ = 0.125	No (non‐inferiority)	3 months: 63.3 (18.6) (PGWBI) n.a. (n.a.) (SMBQ) 6 months: 63.3 (14.5) (PGWBI) n.a. (n.a.) (SMBQ) 12 months: 63.5 (16.8) (PGWBI) n.a. (n.a.) (SMBQ)	3 months: 63.4 (21.5) (PGWBI) n.a. (n.a.) (SMBQ) 6 months: 65.9 (19.9) (PGWBI) n.a. (n.a.) (SMBQ) 12 months: 64.9 (21.9) (PGWBI) n.a. (n.a.) (SMBQ)	All timepoints: Group × time interaction: All *p* > .05
Triguero‐Mas et al. (2017)	I1: Green environment I2: Blue environment	Urban environment	Mood	Mood: POMS	n.a.	n.a.	n.a.	n.a.	Group × time interaction; regression models	Group × time interaction: I1 vs. C: *p* < .01** I2 vs. C: *p* < .01**	n.a.	Yes	n.a.	n.a.	n.a.
Tsunetsugu et al. (2007)	Forest bathing (walking then watching)	Urban environment (walking then watching)	Affective states Stress/distress	Affective state: SD (calm − roused), SD (comfortable − uncomfortable) (Osgood et al., 1957) Stress‐refresh: SRFT (Mackay et al., 1978)	n.a.	n.a.	n.a.	n.a.	Between‐group comparison of change scores; Wilcoxon signed rank test Pre–post comparison within groups	Between‐group comparison of changes scores: *p* < .05* (SD, I calmer after walking) *p* < .01** (SD, I calmer after watching) *p* < .05* (SD, I more comfort after walking) *p* < .01** (SD, I more comfort after watching) *p* < .05* (SRFT, I more refreshed after walking) *p* < .01** (SRFT, I more refreshed after watching) Pre–post comparison: I: *p* < .05* (SD, calmer after watching) C: *p* < .01** (SD, more roused after walking) C: *p* < .05* (SD, more roused after watching) I: *p* < .05* (SD, more comfort after watching) C: *p* < .05* (SD, less comfort after walking) C: *p* < .01** (SD, less comfort after watching) I: *p* > .05 (SRFT) C: *p* < .05* (SRFT, less refreshed after walking) C: *p* < .05* (SRFT, less refreshed after watching)	n.a.	Yes	n.a.	n.a.	n.a.
Van Den Berg and Custers (2011)	Stressful task, then gardening	Stressful task, then reading nature—magazines	Affective states	Affective state: PANAS (PA, NA)	PA: 3.8 (0.5) NA: 1.4 (0.5)	PA: 3.5 (0.5) NA: 1.5 (0.5)	PA: 3.7 (0.5) NA: 1.2 (0.4)	PA: 3.2 (0.7) NA: 1.4 (0.4)	Group × time interaction; rmANOVA	Group × time interaction: *p* < .05* (PA) *p* > .05 (NA)	Group × time interaction: *η* ^2^ = .13 (PA)	Partial	n.a.	n.a.	n.a.
Vujcic et al. (2017)	Horticulture therapy	Occupational + art therapy	Depression Anxiety Stress/distress	Depression, anxiety, stress: DASS‐21	n.a.	n.a.	n.a.	n.a.	Group × time interaction; ANOVA	Group × time interaction: *p* = .309 (D) *p* = .277 (A) *p* < .027* (S)	Group × time interaction: *η* ^*2*^ = .04 [n.a.] (D) *η* ^*2*^ = .04 [n.a.] (A) *η* ^*2*^ = .16 [n.a.] (S)	Partial superiority (non‐inferiority)	n.a.	n.a.	n.a.
Wang et al. (2021)	Nature walk in park	Indoor walk	Mood	Mood: Bf‐S	n.a.	n.a.	n.a.	n.a.	Group × time interaction; multi‐level modeling	Group × time interaction: *p* < .05*	Group × time interaction: *ß* = −.31, SE = .14	Yes	n.a.	n.a.	n.a.
Yen and Huang (2024)	Nature contact	C1: VR nature contact C2: No intervention	Psychological well‐being	Well‐being: WHOQOL‐BREF (Yao et al., 2002)	3.23 (0.6)	C1: 3.6 (0.6) C2: 3.2 (0.7)	0.21 [−.39, −.03]^g^	C1: 0.03 [−.17, .23]^g^ C2: −0.21 [−.39, −.03]^g^	Group × time interactionGEE	Group × time interaction: *p* = .247	n.a.	No	n.a.	n.a.	n.a.

Abbreviations: A, anxiety; ANOVA, analysis of variance; BAT, Burnout Assessment Tool; BDI‐FS, Beck Depression Inventory—Fast Screen; BDI‐I, Beck Depression Inventory; BDI‐II, Beck Depression Inventory II; Bf‐S, Befindlichkeitsskala; C, control group; CAS‐PA, 10‐item Chinese Affect Scale—positive affect subscale; CBT, cognitive behavioral therapy; CC, control condition; CI, confidence interval; CORE‐10, 10‐item Clinical Outcomes in Routine Evaluation; D, depression; DASS‐21, 21‐item Depression Anxiety Stress Scale; DT, distress thermometer; FS, Feeling Scale; Flour. Sc., Flourishing Scale; GAD‐7, 7‐item Generalized Anxiety Disorder Scale; GDS‐15, 15‐item Geriatric Depression Scale; GEE, generalized estimating equation; GLM, general linear model; HADS, Hospital Anxiety and Depression Scale; HADS‐A, Hospital Anxiety and Depression Scale—anxiety subscale; HADS‐D, Hospital Anxiety and Depression Scale—depression subscale; I, intervention group; K6, Short‐form Kessler‐6 Psychological Distress Scale; LMM, linear mixed model; MANOVA, multivariate analysis of variance; MBI‐D, Maslach Burnout Inventory—German version; MBI‐GS, Maslach Burnout Inventory—General Survey; MBSR, Mindfulness‐based Stress Reduction; MFS, Mental Fatigue Scale; Mini‐Z, questionnaire on work‐related burnout symptoms, modified from the Minimizing Error Maximizing Outcome (MEMO) questionnaire; n.a., not applicable; NNBT, Nacadia® nature‐based therapy; n.s., not significant; OLBI, Oldenburg Burnout Inventory; PAAS, Physical Activity Affective Scale; PANAS (PA, NA), Positive and Negative Affect Schedule (positive affect, negative affect); PGWBI, Psychological General Well‐Being Index; PHQ‐9, Patient Health Questionnaire; POMS, Profile of Mood States; POMS‐F, Profile of Mood States—fatigue subscale; POMS‐V, Profile of Mood States—vigor subscale; PSS‐10, 10‐item Perceived Stress Scale; PWB, Ryff's Scale of Psychological Well‐being; PWBI, Personal Well‐being Index; PWI‐C, Personal Well‐being Index—Chinese version; rmANOVA, repeated‐measures analysis of variance; EBF, Erholungs–Belastungs–Fragebogen (Recovery‐Stress Questionnaire); S, stress; SAS, Self‐rating Anxiety Scale; SASRQ, Stanford Acute Stress Reaction Questionnaire; SCL‐90, Symptom Check List; SCS‐SF, Short‐form Self‐Compassion Scale; SD (calm − roused), semantic differential between calm and roused; SD (comfortable − uncomfortable), semantic differential between comfortable and uncomfortable; SDS, Self‐rating Depression Scale; SF‐12, Short‐form 12‐item Health Questionnaire; SF‐8, Short‐form 8‐item Health Questionnaire; SHS, Subjective Happiness Scale; SMBQ, Shirom–Melamed Burnout Questionnaire; SPANE, Scale of Positive and Negative Experience; SRFT, Stress–Refresh Feeling Test; STAI, State‐Trait Anxiety Inventory; SWLS, Satisfaction with Life Scale; TAU, treatment as usual; TMD, Total Mood Disturbance Score; UCLA, UCLA Loneliness Scale; VR, virtual reality; WEMWBS, Warwick Edinburgh Mental Well‐being Scale; WHO‐5, The WHO‐Five Well‐being Index; WHOQOL‐BREF, Short‐form World Health Organization Quality‐of‐Life Scale.

^a^ Values presented as median (interquartiles).

^b^ Values presented as estimated marginal means (standard error).

^c^ Values presented as mean (standard error).

^d^ Values presented as median.

^e^ Represents change scores.

^f^ The values reported for K6 represent percentages of participants who met or exceeded the clinical threshold on the Kessler‐6 Psychological Distress Scale (score ≥13), analogous procedure for the emotion‐related questions.

^g^ Represents mean difference [confidence interval].

^h^ Represents the total mood disturbance score (TMD).

^i^ Repeated measures ANOVA—not specified in the manuscript, but inferred from the available information.

* *p* < .05.

** *p* < .01.

*** *p* < .001.

### Study quality assessment

The risk of bias in the included studies was assessed by three independent reviewers (M.D.B., E.H., D.H.) using the revised Cochrane Risk of Bias tool for RCTs (RoB 2) (Sterne et al., 2019). Each study was assigned an overall risk of bias judgment, categorized as *low risk*, *some concerns*, or *high risk*, in accordance with RoB 2 guidelines. The following domains were evaluated: (1) bias arising from the randomization process, (2) bias due to deviations from intended interventions, (3) bias due to missing outcome data, (4) bias in measurement of the outcome, and (5) bias in selection of the reported result. Depending on the randomization design, the standard RoB 2 tool was used for parallel‐group trials, and the crossover‐specific extension was applied where appropriate. The extension also considered a domain for bias arising from period and carryover effects. Details of the individual assessments are presented in Tables 3 and 4.

**TABLE 3:** Risk of bias assessment (RoB2) of parallel‐group trials.

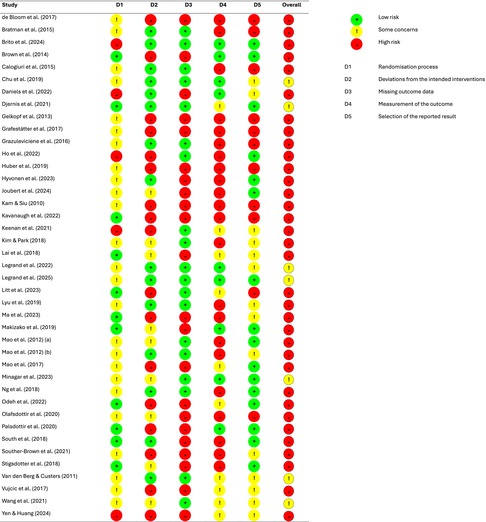

**TABLE 4:** Risk of bias assessment (RoB2) of crossover trials.

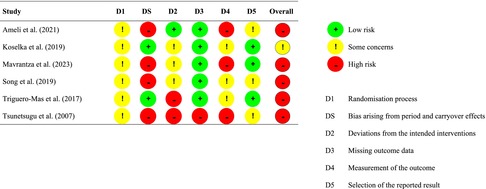

### Synthesis method

A comprehensive narrative synthesis was conducted for all included studies, following the guidelines outlined by (Popay et al., 2006). In line with step 1 (“developing a theory”) of this guidance, the synthesis process was guided by the conceptual framework described in the introduction, which informed the selection of outcomes, the categorization of interventions, and the interpretation of findings. Due to substantial heterogeneity in study designs, intervention types, populations, outcome measures, and statistical reporting, a meta‐analysis was not feasible. Descriptive quantitative analysis was carried out using Microsoft Excel. Findings were organized by outcome domains (e.g., depression, anxiety, stress) and interpreted in relation to study design (superiority vs. non‐inferiority), intervention type, and sample characteristics.

## RESULTS

Results are presented in relation to the conceptual framework described in the introduction which outlines the theoretical mechanisms linking NBIs to mental health outcomes.

### Screening process

**FIGURE 2:**
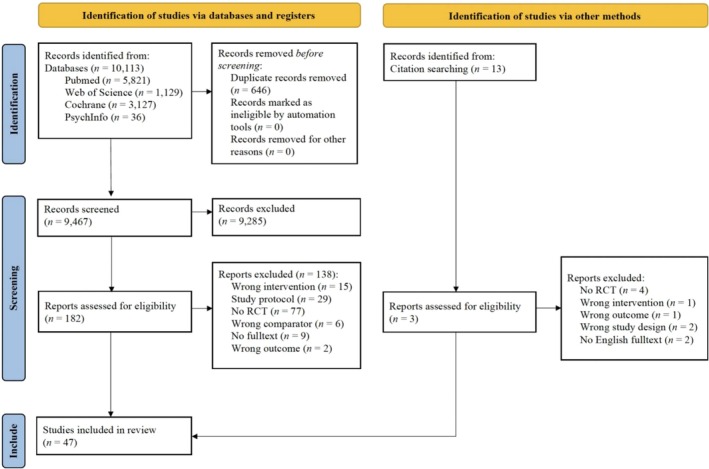
Flowchart of study selection. *Source*: Page et al. (2021). This work is licensed under CC BY 4.0. To view a copy of this license, visit https://creativecommons.org/licenses/by/4.0/.

A total of 10,113 articles were identified through four databases (PubMed: *n* = 5821; Cochrane Library: *n* = 3127; Web of Science: *n* = 1129; PsycINFO: *n* = 36). After removing 646 duplicates, 9467 records remained for screening. Following title and abstract screening, 9285 records were excluded for not meeting the inclusion criteria. A total of 182 full‐text articles were assessed for eligibility, of which 44 studies met the final inclusion criteria. In accordance with our preregistered protocol, we subsequently screened the reference lists of all included studies. This additional step led to the identification of 13 further studies, of which three met the eligibility criteria and were included in the review. As a result, a total of 47 studies were included in the final review. The PRISMA flowchart illustrating the complete study selection process is presented in Figure 2.

### Study characteristics

A total of 47 studies published between 2010 and 2025 were included in this review. Altogether, these studies involved 3792 participants, including 1806 assigned to intervention groups and 2116 to control groups (and *n* = 130 in crossover design studies), with individual sample sizes ranging from 12 to 342 (see Table 1) and varying age and gender distributions. The studies were conducted in various countries, including China, Scandinavia, the United States, Portugal, the United Kingdom, and France. All included studies followed an RCT design. Within this group, a subset (*n* = 6) used a crossover randomization approach, allowing participants to receive both the intervention and the control conditions in a sequential, counterbalanced order.

The NBIs assessed encompassed structured activities such as nature walks, outdoor calisthenics, green exercise programs, and horticultural therapy. The most frequently investigated interventions were nature walks (*n* = 16) and horticultural therapy (*n* = 10). Comparator conditions varied across studies, most commonly consisting of urban‐based or indoor activities (e.g., urban walking, *n* = 11; indoor exercise, *n* = 3), as well as TAU (*n* = 3) and waitlist controls (*n* = 5). Mental health outcomes were primarily assessed by validated measures using self‐reports and including domains, such as depression, anxiety, mood, affect, and burnout. A summary of study characteristics is presented in Table 1. The outcome measures and results are shown in Table 2.

### Quality assessment

In the overall judgment, the risk of bias assessment rated none of the 47 included studies as having a low risk of bias. Eight studies were rated with some concerns (Chu et al., 2019; Djernis et al., 2021; Koselka et al., 2019; Legrand et al., 2022, 2025; Minagar et al., 2023; Van Den Berg & Custers, 2011; Wang et al., 2021) and 39 studies to be of high risk of bias (Ameli et al., 2021; Bratman et al., 2015; Brito et al., 2024; Brown et al., 2014; Calogiuri et al., 2015; Daniels et al., 2022; De Bloom et al., 2017; Gelkopf et al., 2013; Grafetstätter et al., 2017; Grazuleviciene et al., 2016; Ho et al., 2022; Huber et al., 2019; Hyvönen et al., 2023; Joubert et al., 2024; Kam & Siu, 2010; Kavanaugh et al., 2022; Keenan et al., 2021; Kim & Park, 2018; Lai et al., 2018; Litt et al., 2023; Lyu et al., 2019; Ma et al., 2023; Makizako et al., 2019; Mao et al., 2017; Mao, Cao, et al., 2012; Mao, Lan, et al., 2012; Mavrantza et al., 2023; Ng et al., 2018; Odeh et al., 2022; Olafsdottir et al., 2020; Pálsdóttir et al., 2020; Song et al., 2019; Souter‐Brown et al., 2021; South et al., 2018; Stigsdotter et al., 2018; Triguero‐Mas et al., 2017; Tsunetsugu et al., 2007; Vujcic et al., 2017; Yen & Huang, 2024). Notably, the second (deviations from the intended interventions), third (missing outcome data), and fourth (measurement of the outcome) domains represented an increased risk of bias, whereby the first (randomizing process) and fifth (selection of the reported result) domains showed the lowest risk of bias. Studies with substantial dropout rates that did not address how missing data were handled (e.g., through imputation, sensitivity analyses, or intention‐to‐treat principles) were rated as high risk in domain 3. A major source of bias was identified in domain 4 (measurement of the outcome): Most studies relied on self‐report questionnaires without blinding, which, given the subjective nature of mental health outcomes, increases the risk of detection bias through expectancy or social desirability effects. Without objective measures or adequate blinding, internal validity is weakened. These patterns indicate recurring methodological limitations, particularly in outcome measurement and handling of missing data. Detailed risk‐of‐bias judgments are shown in Tables 3 and 4.

### Intervention effects on mental health

Due to high heterogeneity in study designs, intervention types, and outcome measures, no meta‐analysis was conducted. Instead, findings are presented narratively and categorized by outcome domains. Across studies, NBIs generally demonstrated beneficial short‐term effects on mental health, particularly in domains such as depression, mood, and affect. Long‐term effects were assessed less frequently and showed more variability. The following sections summarize the effects of NBIs across key mental health outcomes and intervention types.

#### Depression

Fifteen studies assessed depressive symptoms using various validated instruments (see Table 2).

Overall, most studies used superiority designs and reported improvements favoring NBIs (*p* < .001–.04), most commonly in comparison to urban environments, waitlist controls, or no intervention control (Chu et al., 2019; Gelkopf et al., 2013; Grafetstätter et al., 2017; Ho et al., 2022; Kam & Siu, 2010; Minagar et al., 2023; South et al., 2018). Reported effect sizes ranged from small to medium (e.g., η^2^ = .11; Gelkopf et al., 2013), suggesting meaningful symptom reductions.

Four studies did not find significant differences between groups (Hyvönen et al., 2023; Lai et al., 2018; Makizako et al., 2019; Ng et al., 2018), although within‐group pre–post improvements were found in three studies (all *p* < .001; Hyvönen et al., 2023; Kim & Park, 2018; Makizako et al., 2019).

Among the three non‐inferiority trials, NBIs showed no significant differences in depression outcomes compared to active control groups such as art therapy and TAU (Odeh et al., 2022; Pálsdóttir et al., 2020; Vujcic et al., 2017).

#### Mood

Eleven studies assessed mood, primarily using the Profile of Mood States (POMS; McNair & Doppleman, 1971) and the Befindlichkeitsskala (Bf‐S; Abele‐Brehm & Brehm, 1986) (see Table 2). Eight superiority trials reported positive effects of NBIs on mood (Legrand et al., 2025; Lyu et al., 2019; Mao et al., 2017; Mao, Cao, et al., 2012; Mao, Lan, et al., 2012; Song et al., 2019; Triguero‐Mas et al., 2017; Wang et al., 2021). Three studies found significant interaction effects favoring NBIs, indicating greater mood improvements compared to control conditions over time (Legrand et al., 2025; Triguero‐Mas et al., 2017; Wang et al., 2021; *p* = .003 to <.05). Effect sizes, where reported, were large (e.g., *d* = .96 and *d* = −.87 in Legrand et al., 2025; β = −.31 in Wang et al., 2021). Grafetstätter et al. (2017) reported marginal significance (*p* = .05 and *p* = .06).

Two studies reported significant pre–post improvements within the NBI group only, while the control group either worsened or showed no significant change (Lyu et al., 2019; Mao, Cao, et al., 2012). In contrast, two other superiority trials found no significant intervention effect (Grafetstätter et al., 2017; Ma et al., 2023).

The only non‐inferiority trial (Odeh et al., 2022) reported comparable mood improvements in both NBI and control group (art therapy), with moderate to large pre–post effect sizes (*d* = −.81 and −.60).

#### Anxiety

Fourteen studies assessed anxiety using validated screening measures (see Table 2).

Seven of 11 superiority trials showed significant effects favoring NBIs (all *p* < .05), typically versus control activities or waitlist (Bratman et al., 2015; Grafetstätter et al., 2017; Ho et al., 2022; Joubert et al., 2024; Kam & Siu, 2010; Koselka et al., 2019; Song et al., 2019). For instance, Ho et al. (2022), comparing nature contact during lunch breaks with usual lunch breaks, found significantly lower anxiety levels in the NBI group (*p* = .01). In contrast, three trials found no effects (Litt et al., 2023; Ng et al., 2018; South et al., 2018), and one study reported improvements only within the NBI group but not in the control group (Kim & Park, 2018).

All three non‐inferiority trials showed no group differences (Odeh et al., 2022; Pálsdóttir et al., 2020; Vujcic et al., 2017), indicating that NBIs were as effective as the respective control condition (art therapy, occupational therapy combined with art therapy). Although Odeh et al. (2022) found moderate within‐group effects for the NBI group (*d* = −.45), another trial reported significant improvements only in the NBI group (*p* = .005) (Pálsdóttir et al., 2020).

Overall, the majority of superiority trials suggest that NBIs can effectively reduce anxiety symptoms. Evidence from non‐inferiority trials indicates that NBIs are at least as effective as established treatments.

#### Psychological well‐being

Thirteen studies assessed well‐being and related quality of life constructs using validated scales (see Table 2).

Among the superiority trials, six of 11 studies reported significant group × time interaction effects or between‐group differences in change scores favoring NBIs (*p* = .001 to <.05) (Brown et al., 2014; De Bloom et al., 2017; Ho et al., 2022; Huber et al., 2019; Keenan et al., 2021; Souter‐Brown et al., 2021). Ho et al. (2022) found a significant group X interaction for well‐being (*p* = .005). Souter‐Brown et al. (2021) observed a significant between‐group difference in change scores on the flourishing scale (*p* = .001), but only in comparison with an urban plaza control group, not with the no‐intervention group. Huber et al. (2019) reported mixed results: One NBI group (green exercise) showed no group × time interaction effect (*p* = .057), while the combined intervention (green exercise and balneotherapy) reached significance in favor of NBI (*p* < .001). De Bloom et al. (2017) reported improvements limited to fatigue, with small to moderate effects (*d* = .29–.54). The other five superiority trials did not report significant effects (Kam & Siu, 2010; Lai et al., 2018; Makizako et al., 2019; Ng et al., 2018; Yen & Huang, 2024).

Both non‐inferiority trials (Odeh et al., 2022; Stigsdotter et al., 2018) showed no significant interaction effects, suggesting that NBIs were at least as effective as the respective comparison treatments. Additionally, Stigsdotter et al. (2018) observed significant pre–post improvements in both NBI groups (*p* < .001) and the control group (*p* < .05), with small effect sizes (e.g., *η* ^2^ = .144, *ω* ^2^ = .125).

#### Affective states

Fourteen superiority trials assessed affect using validated scales of positive and negative affect (see Table 2).

Five studies reported significant effects in favor of NBIs (*p* = .001 to <.05), typically versus indoor activity, urban settings, waitlist controls, or no intervention control (Bratman et al., 2015; Keenan et al., 2021; Legrand et al., 2022; Olafsdottir et al., 2020; Tsunetsugu et al., 2007). Reported effects were large (e.g., partial *η* ^2^ = .29–66; *d* = .83) (Keenan et al., 2021; Legrand et al., 2022). Effects were more consistently observed for positive affect than for reductions in negative affect. Three studies reported no effects (Brito et al., 2024; Grazuleviciene et al., 2016; Souter‐Brown et al., 2021), and six studies found condition related results. For example, Calogiuri et al. (2015) observed effects only after adjusting for baseline values, while Ho et al. (2022) and Koselka et al. (2019) found differences in change scores without interaction effects. Selective effects were also observed on specific affect items (South et al., 2018), and one study found an improvement in positive affect but not in negative affect when comparing gardening to reading (Van den Berg & Custers, 2011). Although Mavrantza et al. (2023) found no overall group × time interaction effect, positive affect during exercise sessions was significantly higher in the NBI group than in indoor or virtual conditions (*p* = .003–.014; *η* ^2^ = .20).

#### Burnout

Six studies assessed burnout‐related outcomes using validated instruments (see Table 2).

Four were superiority trials with mixed findings. Daniels et al. (2022) reported a significant group × time interaction (*p* < .001), comparing nature walks to non‐intervention. Kavanaugh et al. (2022) reported a post‐intervention effect on the Mini‐Z (*p* = .03) that did not remain significant after adjustment for multiple comparisons. Ho et al. (2022) reported a significant effect in favour of NBI when comparing change scores between groups (*p* < .05), while Grafetstätter et al. (2017) found no significant differences between conditions.

Both non‐inferiority trials (Pálsdóttir et al., 2020; Stigsdotter et al., 2018) indicated that NBIs were comparable in effectiveness to the respective control conditions (both *p* > .80), including standard care and CBT.

#### Stress/distress

Twelve studies assessed stress using validated measures (see Table 2).

Among superiority trials, five of 10 reported significant stress reductions following NBIs (all *p* < .05), particularly in forest or garden‐based interventions (Ameli et al., 2021; Ho et al., 2022; Hyvönen et al., 2023; Koselka et al., 2019; Tsunetsugu et al., 2007). Reported effects ranged from small to large, for example, between‐group effects of *r* = .51 (Ameli et al., 2021) and interaction effects of *d* = .42 (Hyvönen et al., 2023). The other five superiority design studies found no significant effects, often when comparing NBIs to indoor or passive controls (Djernis et al., 2021; Grafetstätter et al., 2017; Kam & Siu, 2010; Litt et al., 2023; South et al., 2018). However Djernis et al. (2021) still observed small between‐group effects on perceived stress (Hedges' *g* = .36 vs. MBSR‐indoor; *g* = .34 vs. no intervention). Kam and Siu (2010) found a significant difference in favor of NBI when comparing the change scores of the overall DASS‐21, while the stress subscale did not reach the significance threshold (*p* = .05).

Findings from non‐inferiority trials were mixed. Odeh et al. (2022) found between‐group differences in stress outcomes, indicating NBI effects comparable to art therapy with large within‐group effects (*d* = −.97 for group gardening; *d* = −.85 for art therapy). In contrast, Vujcic et al. (2017) showed superiority of NBI over occupational therapy combined with art therapy in the stress reduction (*p* < .027; η^2^ = .16).

#### Other outcomes

Additional psychological outcomes were addressed in a smaller number of studies (*n* = 6) (see Table 2 for further outcome‐related information). Findings were mixed, suggesting potential benefits of NBIs for certain outcomes. Significant improvements were reported for rumination (Bratman et al., 2015), loneliness in the NBI group compared to TAU (*p* < .001; Chu et al., 2019), PTSD symptoms (*p* = .04; *η* ^2^ = .11; Gelkopf et al., 2013), and happiness (*p* = .036; β = 1.457; Lai et al., 2018). In contrast, Djernis et al. (2021) found no significant effects on self‐compassion with negligible to small between‐group effects (Hedges' *g* = .03 vs. MBSR; *g* = −.37 vs. no intervention), and South et al. (2018) reported no significant effect for the single item “everything an effort” when comparing the NBI to a no intervention control.

### Effects by intervention type

Nature walking was the most frequently studied intervention (*n* = 16), and the large majority reported beneficial effects across mental health outcomes (see Table 2 for further information). For example, Ameli et al. (2021) found a large between‐group reduction in stress/distress (*p* = .02; *r* = .51), and Keenan et al. (2021) reported large group × time interaction effects for well‐being (*ηp* ^2^ = .29) and affect (*ηp* ^2^ up to .66; all *p* < .001). Similarly, Legrand et al. (2022) found a large between‐group effect on positive affect (*d* = .83). Two studies reported no effects (Grazuleviciene et al., 2016; Ma et al., 2023).

Horticultural therapy, greening, and gardening showed more variable findings. Several studies reported significant benefits over routine or social control activities. For example, Van den Berg and Custers (2011) reported a significant improvement in positive affect following gardening compared to reading nature magazines (*p* < .05; η^2^ = .13, medium to large effect). Lai et al. (2018) found a significant group × time interaction for happiness (*p* = .036) with a medium effect (β = 1.457) comparing horticultural therapy and social activities. Vujcic et al. (2017) observed a significant interaction for stress (*p* < .027; η^2^ = .16), indicating a large effect in favor of the NBI. However, multiple studies found no superiority over control conditions, and two non‐inferiority trials indicated comparable effects to active treatments. In one of these, both intervention and control groups showed large within‐group stress reductions (*d* = 0.85–0.97; Odeh et al., 2022).

Nine studies examined forest bathing and other nature contacts (e.g., meetings in a park or visiting a sensory garden). Effects included reductions in depression and psychological distress. For example, Hyvönen et al. (2023) reported small to moderate between group effects for depression *d* = .16 and psychological distress *d* = .42. Two studies found no significant differences (Kavanaugh et al., 2022; Yen & Huang, 2024).

Green exercise (e.g., hiking, calisthenics, and sailing) was examined in six studies (see Table 4) by showing positive effects. Gelkopf et al. (2013) reported medium effects (η^2^ = .11) for both depression and PTSD symptoms. Legrand et al. (2022) found a large effect for positive affect compared with an urban walk (*d* = .83). In contrast, some studies found benefits only when the intervention was combined (Huber et al., 2019) or after statistical adjustment (Calogiuri et al., 2015). One study reported no group differences (Brito et al., 2024). Similarly, Mavrantza et al. (2023) found no significant interaction (*p* = .138; η^2^ = .063) but found significantly higher positive feelings in the NBI group compared to indoor and virtual green exercise during the intervention sessions (*p* = .003 and *p* = .014, respectively).

Two studies compared therapy in a natural environment to therapy indoor or with no intervention. For example, Djernis et al. (2021) found no significant interaction effects for stress, although small between‐group effects were observed (*g* = .34–.37) by examining MBSR in a therapy garden versus indoor MBSR and no intervention. Stigsdotter et al. (2018) investigated NNBT versus CBT and reported no interaction for well‐being, despite moderate overall time effects (η^2^ = .13).

Overall, many studies report significant benefits of NBIs over control groups, particularly for nature walking, forest bathing, and, in some cases, gardening or exercise‐based interventions. However, some studies, especially those involving gardening interventions and comparisons with established therapies, found no or only limited superiority over control conditions.

### Study design and comparisons

Most studies (*n* = 43) used superiority designs (six crossover trials), typically comparing NBIs to urban environments, indoor activities, or waitlists. While superiority trials generally favored NBIs, all non‐inferiority trials (*n* = 4) found NBIs to be as effective as control conditions (CBT, TAU, occupational and art therapy), and in one case even superiority regarding stress reduction compared to occupational and art therapy (Vujcic et al., 2017).

Eight studies compared NBIs to active controls such as CBT, MBSR, or TAU, showing selective but meaningful benefits (*p* = .03 to <.001; see Table 4 for further information). For example, significant effects were found for horticultural therapy over TAU in reducing depression (*p* = .004; Minagar et al., 2023), anxiety (*p* = .017; Joubert et al., 2024), and stress (*p* < .027, *η* ^*2*^ = .16; Vujcic et al., 2017). Nature contact during lunch breaks improved anxiety, depression, and quality of life (all *p* < .05; Ho et al., 2022). In contrast, other trials reported comparable outcomes between NBIs and established therapies, such as Nacadia nature‐based therapy versus CBT (Stigsdotter et al., 2018) and garden‐based versus indoor MBSR (Djernis et al., 2021), with only small between‐group effects (*g* = .34–.37).

Across 14 studies comparing NBIs with no intervention or waitlist controls, findings were mixed but generally support positive mental health effects. Significant improvements were reported for combined green exercise and balneotherapy (*p* = .001; Huber et al., 2019), nature walks (*p* < .001; Daniels et al., 2022), and green exercise on depressive and PTSD symptoms (both *p* = .04; Gelkopf et al., 2013). Reductions in stress, anxiety, and distress were also reported in several trials through horticultural therapy (Kim & Park, 2018), sensory garden use (Souter‐Brown et al., 2021), and nature contact involving a waterfall (Grafetstätter et al., 2017), all *p* < .05. While other studies found no effects (e.g., Djernis et al., 2021) (MBSR in a garden), Litt et al. (2023) (gardening), Yen and Huang (2024) (nature contact), and Ng et al. (2018) (horticultural therapy). Further study information is provided in Tables 1 and 2.

### Setting

A comparison of studies conducted in forest or wilderness settings versus those in urban parks or managed green spaces reveals notable differences in outcomes (see Tables 1 and 2).

Several trials in natural, wild environments showed medium to large effect sizes, including reductions in depression and stress (η^2^ = .11; Gelkopf et al., 2013), large improvements in affect and well‐being (ηp^2^ = .29–.66; Keenan et al., 2021), and large improvements in positive affect (*d* = .83; Legrand et al., 2022).

In contrast, interventions situated in urban or managed nature settings such as parks or botanical gardens showed more mixed results. Some reported significant improvements such as a moderate to large reduction in stress compared with urban walking (*r* = .51; Ameli et al., 2021) and small to moderate improvements in psychological distress (*d* = .42; Hyvönen et al., 2023). However, effects on depression in the same park‐based intervention were small (*d* = .16; Hyvönen et al., 2023). Larger benefits were found in Legrand et al. (2025) for green exercise compared to a regular class (*d* = .96 for vigor; *d* = −.87 for fatigue), whereas several other studies found no significant differences.

Overall, urban green space interventions can provide benefits, but their effects are generally smaller, less consistent, and more context dependent than those in wilderness settings.

### Intervention format

Most studies used group‐based NBIs (*n* = 33), followed by individually delivered formats (*n* = 12) and two mixed‐format interventions (see Table 1).

Group‐based interventions typically involved structured programs such as forest bathing, horticultural therapy, or guided nature walks and were associated with improvements across depression, anxiety, stress, and well‐being outcomes. Reported effects ranged from small to large. For example, Gelkopf et al. (2013) found medium effects for depression and posttraumatic stress, both η^2^ = .11, while Djernis et al. (2021) reported small‐to‐moderate effects for stress and self‐compassion (*g* = .34–.37).

Individual delivered interventions also demonstrated beneficial effects, particularly for stress and affect. Ameli et al. (2021) reported medium to large effects on well‐being (*r* = .51–.61), De Bloom et al. (2017) found small to moderate effects on fatigue (*d* = .29–.54) and Olafsdottir et al. (2020) demonstrated a large effect on positive affect (*η* ^2^ = .20).

The two mixed‐format studies yielded consistent results, including superiority over an urban environment (all *p* < .01) and non‐inferiority in another trial comparing against CBT. See Table 2 for further outcome‐related results.

### Frequency, duration, and intensity

The included studies varied widely in the reporting and structuring of frequency, duration, and intensity of NBIs, ranging from single sessions to multi‐week programs and from passive nature contact to moderate physical activity, making it methodologically inappropriate to draw generalizable conclusions about dose–response relationships.

### Follow‐up data

Fifteen studies included follow‐up assessments ranging from several days to 14 months (see Table 2). Some studies reported improvements in specific outcomes, such as affect (*p* = .02; Calogiuri et al., 2015), burnout (*p* < .05; Daniels et al., 2022), self‐compassion (*p* = .030; Djernis et al., 2021), and stress recovery (*p* = .002; Grafetstätter et al., 2017) (see Table 4). Longer‐term benefits were also observed for perceived stress (*p* = .019; Ho et al., 2022), psychological distress (*p* < .05; Hyvönen et al., 2023), and well‐being (*p* < .001; Keenan et al., 2021). However, several trials found no significant between‐group differences at follow‐up for depression, anxiety, or quality of life (e.g., Huber et al., 2019; Ma et al., 2023; Ng et al., 2018; Pálsdóttir et al., 2020; Stigsdotter et al., 2018). Overall, positive effects were most often seen in short‐ to medium‐term follow‐ups, while longer‐term findings were mixed.

### Patterns and moderators

Across the included studies, nature walks and horticultural therapy were the most frequently investigated NBIs and were consistently associated with improvements in mental health outcomes, particularly in depression, anxiety, and mood (*p* values ranging from <.001 to .91). Reported effect sizes in studies ranged from medium to large, for example, *r* = .51 for stress in Ameli et al. (2021), *d* = .83 for positive affect in Legrand et al. (2022), and β = −.31 for mood in Wang et al. (2021). Interventions conducted in natural outdoor settings generally produced greater effects than those in urban or indoor environments, with reported *p* values ranging from <.001 to .03 and medium to large effect sizes in several trials (e.g., *η* ^2^ = .20 for positive affect in Olafsdottir et al., 2020). Preliminary evidence suggests that intervention duration, format, and participant characteristics (e.g., age, baseline distress) may moderate effectiveness. However, inconsistencies in reporting and study design limited calculating subgroup analyses. Studies with active control groups (*p* < .001 to *p* = .74) and those with passive comparators (*p* < .001 to *p* = .93) demonstrated similarly wide ranges of effects, with reported effect sizes ranging from negligible (*η* ^2^ < .01; Ma et al., 2023) to large (*d* = −.87; Legrand et al., 2025), suggesting that the type of control condition may not systematically influence outcomes.

### Robustness of the evidence

The overall robustness of the evidence is limited by heterogeneity in study designs, intervention formats, and outcome measurements. While all included studies were RCTs, methodological quality varied considerably. Common limitations included insufficient reporting of randomization procedures, lack of blinding, small sample sizes, and incomplete follow‐up data. The diversity of outcome measures and the inconsistent reporting of effect sizes and confidence intervals hindered comparability across studies. Moreover, only a minority of studies provided detailed subgroup analyses or moderator assessments, which restrict conclusions about differential effectiveness across populations or settings. Despite these limitations, the direction of effects was largely consistent in favor of NBIs. The convergence of findings across intervention types, settings, and populations provides moderate confidence in the reliability of the observed effects. However, future research should aim to improve methodological rigor and standardize outcome reporting to strengthen the evidence base.

## DISCUSSION

This systematic review synthesized evidence from 47 RCTs examining the effectiveness of NBIs in improving mental health outcomes in adults. These interventions included nature walks, green exercise (e.g., hiking, running, sailing, and calisthenics in the park), horticultural therapy, gardening, forest bathing, and group meetings in the park. The mental health outcomes included depression, mood, anxiety, psychological well‐being, affective states, burnout, stress, and other outcomes.

### Summary of findings

Of the 47 RCTs included in this review, 17 studies (36.2%) reported statistically significant between‐group effects in favor of NBIs on at least one mental health outcome. An additional 15 studies (31.9%) demonstrated partial or conditional effects, such as significant results for specific subdomains, selected outcomes, or within‐group changes only. In contrast, 11 studies (23.4%) reported no evidence of superiority or non‐inferiority of NBIs compared with the respective control conditions. Taken together, approximately 68.1% of the studies indicated some mental health benefit associated with NBIs, with considerable variability in effect strength, outcome specificity, and methodological quality.

While short‐term improvements in mental health outcomes were frequently observed, only 16 of the 47 included studies conducted follow‐up assessments, with observation periods ranging from 5 days to 14 months. Among these, eight trials (50%) reported sustained between‐group effects in favor of NBIs in at least one outcome at follow‐up. The remaining studies either showed no significant effects across timepoints or reported only within‐group changes without corresponding between‐group differences. These findings underscore the need for more rigorous long‐term evaluations to determine the lasting impact of NBIs on psychological health.

### Comparison conditions

How do NBIs compare to other therapeutic interventions or no intervention in improving mental health (RQ2)? NBIs were evaluated against both active and passive comparators. NBIs were generally more effective than passive controls and showed comparable effectiveness (non‐inferiority) to established treatments. There was no consistent evidence that NBIs are superior to these treatments, but they appear to be viable alternatives or complements.

Eight studies employed active control conditions, including CBT, TAU, or occupational and art therapy. Among these, three demonstrated non‐inferiority, two reported significant superiority of NBIs on at least one outcome, and one showed partial effects. In contrast, 14 studies used passive control groups (e.g., waitlist, no treatment). While some of these showed favorable effects, including two studies with significant between‐group differences and two with partial or within‐group improvements, others yielded no clear evidence of benefit. These results indicate that NBIs may be comparably effective to standard interventions in selected domains, but further high‐quality head‐to‐head trials are needed.

### Mental health outcomes

Which mental health outcomes are influenced by NBIs (RQ3)? The strongest and most consistent effects were observed for depression, anxiety, and mood. Moderate evidence was found for stress reduction and affective improvements, while findings for well‐being and burnout were more mixed.

Depression and anxiety were the most frequently assessed outcomes across studies. Of 15 trials evaluating depression, 11 (73.3%) demonstrated at least some indication of benefit from NBIs, including seven trials with statistically significant between‐group effects, three reporting non‐inferiority, and several showing partial or within‐group improvements. Similarly, among 14 studies assessing anxiety, 10 (71.4%) reported positive outcomes, including three with non‐inferiority relative to comparators.

Stress reduction also emerged as a consistent target, with 12 studies assessing stress or psychological distress. Of these, five trials (41.7%) reported partial between‐group effects, and one demonstrated non‐inferiority. For burnout, six studies were included, of which one reported significant effects, two reported partial effects, and two showed non‐inferiority compared to active controls.

Psychological well‐being was addressed in 13 studies, with three reporting effects, three reporting partial effects, and seven yielding no effect or only within‐group effects. Affective states were examined in 14 studies, with five studies reporting significant effects, three studies reporting no improvements in mood or affect, and six studies reporting at least partial effects, typically in relation to natural versus urban settings. These findings highlight the potential of NBIs to enhance psychological functioning, though there were indications that effect strength and consistency may differ depending on the outcome assessed and the methodological quality of individual studies.

### Intervention types

What types of NBIs improve mental health outcomes (RQ1)? The effectiveness of NBIs varied by intervention modality. The most consistent evidence was found for nature walks, horticultural therapy, and forest bathing, with nature walks being the most frequently studied and showing the most robust effects. Interventions involving active engagement with nature (e.g., gardening) tended to yield more consistent benefits than passive exposure.

Among 14 studies evaluating horticultural therapy or gardening, four reported statistically significant effects, four yielded partial benefits, four reported no effect, and two reported non‐inferiority. These interventions frequently involved direct interaction with nature and were among the most consistently favorable approaches. In line with recent conceptual work, this supports the notion that nature‐engaging activities may elicit greater psychological benefits than mere passive exposure.

Forest bathing and other nature contact was assessed in nine studies. Seven studies reported significant results, but no consistent pattern of superiority emerged across outcomes or comparator groups. Overall, while the heterogeneity in study design limits direct comparison, interventions involving active, multisensory engagement with natural settings (e.g., gardening, horticulture) appear more robust than those emphasizing movement alone or passive exposure.

Does including physical activity affect outcomes (RQ7)? Sixteen studies investigated nature walks, with outcomes more heterogeneous. Fourteen studies demonstrated significant effects; among the five studies assessing green exercise (e.g., hiking or calisthenics), one reported a significant effect, and several showed marginal or conditional improvements. However, physical activity alone does not fully explain the benefits, suggesting that interaction with nature itself contributes independently.

### Frequency, duration, and intensity

How do frequency, duration, and intensity influence effectiveness (RQ5)? Intervention frequency and duration varied widely across studies, ranging from single‐session exposures to structured multi‐week programs. No clear dose–response relationship could be established due to high heterogeneity and inconsistent reporting. However, single‐session interventions appeared less effective than multi‐session programs.

Notably, 10 studies implemented NBIs as a single‐session intervention: Among these, only one yielded partial evidence of effectiveness. This suggests that one‐time nature exposure may be insufficient to elicit robust or sustained mental health benefits.

Although several multi‐session studies demonstrated favorable outcomes, the considerable heterogeneity in frequency, session length, and intervention duration precluded the identification of clear dose–response patterns. Some effective interventions involved weekly sessions of 30 min or longer, while others used higher frequency or longer durations without a consistent association with outcome strength. These findings underscore the need for future trials to systematically vary and report intervention dosage parameters to enable more precise conclusions about optimal intensity and frequency.

### Natural vs. urban settings

Does the setting influence outcomes (RQ6)? Interventions conducted in natural or wilderness environments generally showed stronger and more consistent effects than those in urban green spaces. Fourteen studies were conducted in natural or wilderness environments such as forests or mountains. Among these, all reported at least one statistically significant mental health outcome. In contrast, 14 studies were carried out in urban parks or managed green spaces; of these, six reported significant effects and four demonstrated partial effects, and four no effects. These findings suggest that while both settings can support psychological benefits, the type and quality of natural environment—such as biodiversity, sensory input, and perceived naturalness—may moderate outcomes and merit further investigation.

### Intervention format

Does the intervention format influence outcomes (RQ6)? Both group‐based and individual formats were effective, with group interventions potentially offering additional social benefits. Most studies (*n* = 33) implemented NBIs in a group‐based format, such as forest bathing sessions, horticultural therapy groups, or guided nature walks. Of these, four studies demonstrated statistically significant effects, three reported non‐inferiority compared to active controls, and six yielded partial improvements. Three studies reported no effect, and one showed within‐group improvements only.

Twelve studies used individually delivered interventions. Among these, five reported a significant between‐group effect and five yielded partial benefits. Two additional studies employed mixed formats, combining individual and group elements; both showed positive trends but lacked consistent superiority across outcomes.

These findings suggest that both group‐based and individual NBIs can be effective, though group interventions may offer additional benefits through structured guidance, social interaction, or shared engagement with nature. Further research is needed to directly compare delivery formats and disentangle the contribution of social versus environmental components.

### Methodological quality and risk of bias

How robust is the methodological quality of the evidence (RQ4)? The overall methodological quality of included studies was limited, with the majority of studies rated as having a high risk of bias, particularly due to lack of blinding, reliance on self‐report measures, and incomplete data reporting.

According to the risk of bias assessment, none of the 47 trials met criteria for low risk across all domains. Thirty‐nine studies (83%) were rated as having a high risk of bias, particularly due to insufficient reporting of randomization procedures, lack of blinding, missing outcome data, and deviations from the intended interventions. Only eight studies were judged to present some concerns, and none were rated as low risk.

A key source of heterogeneity arose from the diversity of outcome measurement tools. For example, depression was assessed using a range of instruments including the BDI, PHQ‐9, GDS, SDS, HADS, and SCL‐90, among others. Similar variation was observed in the assessment of anxiety, stress, and well‐being. This variability complicates synthesis and limits comparability across studies. Moreover, standardized reporting of outcome data was frequently lacking: 13 studies did not provide pre–post comparison statistics, six failed to report group‐level *p* values, and only 16 of the 47 trials reported effect sizes. This incomplete reporting hinders interpretation and reduces transparency. The predominance of *p* values without effect size estimates limits interpretability, reduces comparability across studies, and hinders conclusions about the significance of findings. Taken together, the substantial heterogeneity in study design, intervention delivery, control conditions, and outcome measurement—combined with generally high risk of bias—limits the strength of the conclusions that can be drawn and underscores the need for more rigorous study designs and standardized reporting in future trials.

### Heterogeneity and meta‐analysis

Substantial heterogeneity was observed across intervention type, duration, frequency, and comparator conditions. NBIs ranged from single‐session exposures to structured multi‐week programs and from passive nature contact to physically demanding activities. Outcome measurement was equally diverse, with different instruments assessing the same constructs and inconsistent follow‐up intervals. These variations, along with inconsistent reporting formats, precluded meaningful subgroup or dose–response analyses and rendered a meta‐analysis inappropriate, in line with Cochrane guidelines. A narrative synthesis was therefore used to summarize findings.

### Strengths and limitations

This review provides a comprehensive synthesis of RCTs evaluating the effects of NBIs on mental health outcomes in adults. A major strength is the exclusive inclusion of RCTs, which enhances internal validity compared to observational studies. The systematic search across four major databases, adherence to PRISMA guidelines, and use of validated risk of bias tools further strengthen methodological rigor. Screening and data extraction were conducted independently by two reviewers, reducing the risk of selection bias.

However, several limitations must be noted. Many included studies had relatively small sample sizes, which may reduce statistical power, and in addition showed a high risk of bias. Moreover, essential statistical information such as effect sizes, confidence intervals, or pre–post data was often not reported. This is problematic, as *p* values alone cannot be interpreted as indicators of effect magnitude. Their dependence on sample size and study power may otherwise lead to misleading impressions of the consistency or strength of effects.

Additionally, four studies were categorized as non‐inferiority trials in this review, based on the use of established treatment comparators such as CBT or combined occupational and art therapy. In these studies, it was not explicitly stated which group was expected to be superior, yet the control interventions were clearly regarded as effective by the original study authors. As such, we interpreted equivalence in outcomes as indicative of non‐inferiority. However, not all of these studies were designed or powered as formal non‐inferiority trials, and their results should therefore be interpreted with appropriate caution. The overall quality of evidence was limited by inconsistent outcome definitions and incomplete follow‐up reporting, and although the review captured a broad international sample, most studies were conducted in high‐ or upper‐middle‐income countries, limiting generalizability to low‐resource settings.

Despite these limitations, the overall consistency in the direction of effects suggests that NBIs represent a promising approach to promoting mental well‐being, particularly through direct nature engagement.

### Theoretical implications

Findings from this review contribute to the theoretical understanding of how NBIs may influence mental health. The observed benefits across diverse outcomes—including mood, anxiety, and well‐being—align with theoretical models such as stress reduction theory and ART, which posit that natural environments facilitate physiological and cognitive recovery from stress and attentional fatigue. Moreover, interventions involving active engagement with nature (e.g., gardening, green exercise) appeared more consistently effective than passive exposure, supporting emerging conceptual frameworks that emphasize the importance of embodied, sensory, and goal‐directed interaction with natural settings. These findings also resonate with affect regulation models, suggesting that nature contact may function as a self‐regulatory resource that enhances emotional stability and resilience. Future research should further investigate these mechanisms, including the roles of multisensory input, social co‐regulation, and perceived connectedness to nature, to refine theory and inform intervention design.

### Co‐benefits of NBIs on mental health and climate action

Our systematic review responds to calls for integrative approaches that simultaneously address the global mental health crisis and environmental degradation. Our work addresses two key SDGs: SDG 3 (Good Health and Wellbeing) and SDG 13 (Climate Action). NBIs represent an accessible, affordable, and low‐risk approach to improving mental health, aligning with SDG 3 (Good Health and Wellbeing). Our systematic review provides evidence of the positive impact of NBIs on several mental health outcomes.

In addition, NBIs support broader sustainability efforts by encouraging access to green spaces in urban areas and fostering nature connectedness (Keenan et al., 2021), which has been linked to pro‐environmental behavior (Mackay & Schmitt, 2019; Martin et al., 2020; Whitburn et al., 2020), and could thereby support progress towards SDG 13 (Climate Action). With this, NBIs appear to have a dual impact that supports both individual health and broader environmental goals. In this way, NBIs align with the “One Health” perspective and contribute to ecological models of health by encouraging pro‐environmental behavior and urban sustainability.

### Implications for public health and policy

These findings are particularly relevant for public health, as NBIs represent a potentially low‐cost, scalable, and accessible intervention format that can complement traditional mental healthcare services. Recent evidence underscores the promise of NBIs within public health systems, particularly in the context of social prescribing and community‐based mental health support (De Bell et al., 2024; Wendelboe‐Nelson et al., 2025). Such approaches offer a pragmatic response to mental health needs in diverse populations—including marginalized groups—while simultaneously addressing environmental and social determinants of health. The results of this review thus not only contribute to the clinical understanding of NBIs but also highlight their value as a policy‐relevant and system‐level strategy for promoting mental well‐being.

### Implications for future research

Future studies should prioritize methodological rigor, including transparent reporting, standardized outcome measures, and the use of adequately powered designs. The high prevalence of incomplete reporting and high risk of bias in existing trials underscores the need for improved adherence to CONSORT guidelines, including preregistration and effect size reporting. Given the heterogeneity observed across intervention types, durations, and settings, future trials should employ more homogeneous designs to enable dose–response analyses and clearer interpretation of active components.

Theoretical mechanisms such as multisensory stimulation, social co‐regulation, and nature connectedness should be explicitly measured to advance explanatory models. Studies should also compare different natural environments (e.g., wilderness vs. urban parks) and clarify the role of setting quality. Long‐term follow‐up assessments are needed, as nearly 70% of studies lacked post‐intervention follow‐up, leaving questions about sustained effects unresolved.

Future implementation research should further explore how NBIs can be integrated into public health infrastructures and what contextual conditions facilitate or hinder their delivery in real‐world settings.

Furthermore, most existing trials were conducted in high‐income settings; research in low‐ and middle‐income countries, especially in diverse ecological and cultural contexts, remains scarce. Finally, head‐to‐head comparisons of NBIs with established psychotherapeutic or pharmacological treatments are warranted to determine whether NBIs can serve as effective stand‐alone or adjunctive options in mental health care.

## CONCLUSION

This systematic review synthesizes evidence from 47 RCTs and suggests that NBIs may offer meaningful mental health benefits, particularly for symptoms of depression, anxiety, and stress. Interventions involving active engagement with nature—such as gardening or horticultural therapy—showed the most consistent effects. However, substantial heterogeneity in intervention design, outcome measurement, and methodological quality limits the strength of current conclusions. While the general trend across trials is encouraging, more robust and consistent research is essential before NBIs can be reliably incorporated into clinical or public health strategies. This review also complements and extends prior systematic reviews by incorporating a broader range of NBI formats, mental health outcomes, and comparator types while restricting inclusion to RCTs published through mid‐2025. In contrast to previous syntheses that focused on specific interventions (Kamioka et al., 2014; Kotera et al., 2022), populations (Cipriani et al., 2017; Gritzka et al., 2020; Rueff & Reese, 2023), or comparisons (e.g., Rueff & Reese, 2023), the present review provides a comprehensive and methodologically focused update of the evidence base. As such, it offers a more integrative perspective on the mental health impacts of NBIs and helps identify key intervention characteristics and research gaps.

## CONFLICT OF INTEREST STATEMENT

All authors declare that there are no conflicts of interest.

## ETHICS STATEMENT

This work is based on previously published studies and does not involve any studies with human participants or animals conducted by the authors. Therefore, ethics approval was not required.

## Supporting information


**Data S1.** Full search term.

## Data Availability

Data sharing not applicable to this article as no datasets were generated or analyzed during the current study.
