# Supplementary material for: The effectiveness of nature‐based interventions on mental health: A systematic review
Source: Appl Psychol Health Well Being. 2026 Jul 2;18(4):e70164. doi: 10.1111/aphw.70164 (PMC13326778; doi:10.1111/aphw.70164)
Supplement: Supplementary file 1 — Data S1. Full search term. [file APHW-18-0-s001.docx]

**Supplementary Material for**

**The Effectiveness of Nature-Based Interventions on Mental Health: A Systematic Review**

**Text 1.** Full search term

**Text 1.** Full search terms

Search terms

**Pubmed**

((("natural"[All Fields] OR "naturally"[All Fields] OR "naturals"[All Fields] OR "nature"[MeSH Terms] OR "nature"[All Fields] OR "nature s"[All Fields] OR "natures"[All Fields]) AND ("based"[All Fields] OR "basing"[All Fields]) AND ("intervention s"[All Fields] OR "interventions"[All Fields] OR "interventive"[All Fields] OR "methods"[MeSH Terms] OR "methods"[All Fields] OR "intervention"[All Fields] OR "interventional"[All Fields])) OR ("gardener"[All Fields] OR "gardeners"[All Fields] OR "gardening"[MeSH Terms] OR "gardening"[All Fields] OR "gardens"[MeSH Terms] OR "gardens"[All Fields] OR "garden"[All Fields]) OR (("green"[All Fields] OR "greened"[All Fields] OR "greening"[All Fields] OR "greenness"[All Fields] OR "greens"[All Fields]) AND ("exercise"[MeSH Terms] OR "exercise"[All Fields] OR "exercises"[All Fields] OR "exercise therapy"[MeSH Terms] OR ("exercise"[All Fields] AND "therapy"[All Fields]) OR "exercise therapy"[All Fields] OR "exercising"[All Fields] OR "exercise s"[All Fields] OR "exercised"[All Fields] OR "exerciser"[All Fields] OR "exercisers"[All Fields])) OR ("relaxation therapy"[MeSH Terms] OR ("relaxation"[All Fields] AND "therapy"[All Fields]) OR "relaxation therapy"[All Fields] OR "ecotherapy"[All Fields]) OR (("natural"[All Fields] OR "naturally"[All Fields] OR "naturals"[All Fields] OR "nature"[MeSH Terms] OR "nature"[All Fields] OR "nature s"[All Fields] OR "natures"[All Fields]) AND ("based"[All Fields] OR "basing"[All Fields]) AND ("walked"[All Fields] OR "walking"[MeSH Terms] OR "walking"[All Fields] OR "walks"[All Fields])) OR ("forest therapy"[MeSH Terms] OR ("forest"[All Fields] AND "therapy"[All Fields]) OR "forest therapy"[All Fields] OR ("forest"[All Fields] AND "bathing"[All Fields]) OR "forest bathing"[All Fields]) OR ("horticultural"[All Fields] OR "horticulturally"[All Fields] OR "horticulture"[MeSH Terms] OR "horticulture"[All Fields]) OR (("communal"[All Fields] OR "communalism"[All Fields] OR "communalities"[All Fields] OR "communality"[All Fields] OR "communally"[All Fields] OR "commune"[All Fields] OR "communes"[All Fields] OR "community s"[All Fields] OR "communitys"[All Fields] OR "residence characteristics"[MeSH Terms] OR ("residence"[All Fields] AND "characteristics"[All Fields]) OR "residence characteristics"[All Fields] OR "communities"[All Fields] OR "community"[All Fields]) AND ("agriculture"[MeSH Terms] OR "agriculture"[All Fields] OR "farming"[All Fields] OR "farm s"[All Fields] OR "farmed"[All Fields] OR "farms"[MeSH Terms] OR "farms"[All Fields])) OR (("natural"[All Fields] OR "naturally"[All Fields] OR "naturals"[All Fields] OR "nature"[MeSH Terms] OR "nature"[All Fields] OR "nature s"[All Fields] OR "natures"[All Fields]) AND ("based"[All Fields] OR "basing"[All Fields]) AND ("arts syndrome"[Supplementary Concept] OR "arts syndrome"[All Fields] OR "arts"[All Fields] OR "art"[MeSH Terms] OR "art"[All Fields])) OR ("environ conserv"[Journal] OR ("environmental"[All Fields] AND "conservation"[All Fields]) OR "environmental conservation"[All Fields])) AND ("mental health"[MeSH Terms] OR ("mental"[All Fields] AND "health"[All Fields]) OR "mental health"[All Fields] OR ("mental disorders"[MeSH Terms] OR ("mental"[All Fields] AND "disorders"[All Fields]) OR "mental disorders"[All Fields] OR ("mental"[All Fields] AND "disorder"[All Fields]) OR "mental disorder"[All Fields]) OR ("depressed"[All Fields] OR "depression"[MeSH Terms] OR "depression"[All Fields] OR "depressions"[All Fields] OR "depression s"[All Fields] OR "depressive disorder"[MeSH Terms] OR ("depressive"[All Fields] AND "disorder"[All Fields]) OR "depressive disorder"[All Fields] OR "depressivity"[All Fields] OR "depressive"[All Fields] OR "depressively"[All Fields] OR "depressiveness"[All Fields] OR "depressives"[All Fields]) OR ("anxiety"[MeSH Terms] OR "anxiety"[All Fields] OR "anxieties"[All Fields] OR "anxiety s"[All Fields]) OR ("stress"[All Fields] OR "stressed"[All Fields] OR "stresses"[All Fields] OR "stressful"[All Fields] OR "stressfulness"[All Fields] OR "stressing"[All Fields]) OR ("affect"[MeSH Terms] OR "affect"[All Fields] OR "affects"[All Fields] OR "affected"[All Fields] OR "affecteds"[All Fields] OR "affecting"[All Fields]) OR ("mental health"[MeSH Terms] OR ("mental"[All Fields] AND "health"[All Fields]) OR "mental health"[All Fields] OR ("mental"[All Fields] AND "well"[All Fields]) OR "mental well being"[All Fields]) OR ("lonelier"[All Fields] OR "loneliness"[MeSH Terms] OR "loneliness"[All Fields]) OR (("negative"[All Fields] OR "negatively"[All Fields] OR "negatives"[All Fields] OR "negativities"[All Fields] OR "negativity"[All Fields]) AND ("affect"[MeSH Terms] OR "affect"[All Fields] OR "mood"[All Fields]))) AND ("RCT"[All Fields] OR ("randomized controlled trial"[Publication Type] OR "randomized controlled trials as topic"[MeSH Terms] OR "randomized controlled trials"[All Fields] OR "randomised controlled trials"[All Fields]) OR ("randomized controlled trial"[Publication Type] OR "randomized controlled trials as topic"[MeSH Terms] OR "randomised controlled trials"[All Fields] OR "randomized controlled trials"[All Fields]))

**Cochrane:**

Nature based intervention OR gardening OR green exercise OR ecotherapy OR nature based walking OR forest bathing OR horticulture OR community farming OR nature based arts OR environmental conservation) AND (mental health OR mental disorder OR depression OR anxiety OR stress OR affect OR mental well being OR loneliness OR negative mood) AND (RCT OR randomized controlled trials OR randomised controlled trials) (Word variations have been searched)

**Web of Science**

Nature based intervention OR gardening OR green exercise OR endotherapy OR nature based walking OR forest bathing OR horticulture OR community farming OR nature based arts OR environmental conservation (All Fields) and mental health OR mental disorder OR depression OR anxiety OR stress OR affect OR mental well being OR loneliness OR negative mood (All Fields) and RCT OR randomized controlled trials OR randomised controlled trials (All Fields)

**PsycInfo**

(Nature based intervention OR gardening OR green exercise OR ecotherapy OR nature based walking OR forest bathing OR horticulture OR community farming OR nature based arts OR environmental conservation) AND (mental health OR mental disorder OR depression  OR anxiety OR stress OR affect OR mental well being OR loneliness OR negative mood) AND (RCT OR randomized controlled trials OR randomised controlled trials)
